# Comprehensive approaches reveal three cryptic species of genus *Nidirana* (Anura, Ranidae) from China

**DOI:** 10.3897/zookeys.914.36604

**Published:** 2020-02-20

**Authors:** Zhi-Tong Lyu, Ke-Yuan Dai, Yao Li, Han Wan, Zhe-Yi Liu, Shuo Qi, Si-Min Lin, Jian Wang, Yu-Long Li, Yang-Jin Zeng, Pi-Peng Li, Hong Pang, Ying-Yong Wang

**Affiliations:** 1 State Key Laboratory of Biocontrol/ The Museum of Biology, School of Life Sciences, Sun Yat-sen University, Guangzhou 510275, China Sun Yat-sen University Guangzhou China; 2 Guangdong Shimentai National Nature Reserve, Qingyuan 513000, China Guangdong Shimentai National Nature Reserve Qingyuan China; 3 Institute of herpetology, Shenyang Normal University, Shenyang 110034, China Shenyang Normal University Shenyang China; 4 Department of Life Science, National Taiwan Normal University, Taipei 116, China National Taiwan Normal University Taipei Taiwan

**Keywords:** bioacoustics, mitochondrial DNA, morphology, *Nidirana guangdongensis* sp. nov., *Nidirana mangveni* sp. nov., *Nidirana xiangica* sp. nov.

## Abstract

Three cryptic species, which were previously reported as *Nidirana adenopleura*, are revealed on the basis of comprehensive approaches. *Nidirana guangdongensis* Lyu, Wan, and YY Wang, **sp. nov.** is distributed in Nanling Mountains and southern Luoxiao Mountains, *Nidirana mangveni* Lyu, Qi, and YY Wang, **sp. nov.** is known from northern Zhejiang, and *Nidirana xiangica* Lyu and YY Wang, **sp. nov.** occurs in Xiangjiang River Basin, while the true *Nidirana adenopleura* is designated from Taiwan Island, northern Fujian, southern Zhejiang, and central Jiangxi. These three new species can be distinguished from all congeners by significant divergences in the mitochondrial 16S and CO1 genes, differences in advertisement calls, and the combination of multiple characteristics. This work indicates that the current records of *Nidirana adenopleura* should be of a species complex composed of multiple species and have clarified the true identity of *N. adenopleura*.

## Introduction

The Music frog genus *Nidirana* Dubois, 1992 was recently reconsidered as a distinct genus based on comprehensive approaches (Lyu et al. 2017). Ten species are currently recognized from subtropical eastern and southeastern Asia: *N. okinavana* (Boettger, 1895) from Yaeyama of southern Ryukyu, and eastern Taiwan; *N. adenopleura* (Boulenger, 1909) from Taiwan and southeastern mainland China; *N. nankunensis* Lyu, Zeng, Wang, Lin, Liu, & Wang, 2017 from Mt Nankun of Guangdong; *N. yaoica* Lyu, Mo, Wan, Li, Pang, & Wang, 2019 from Mt Dayao of Guangxi; *N. hainanensis* (Fei, Ye, & Jiang, 2007) from Mt Diaoluo of Hainan; *N. leishanensis* Li, Wei, Xu, Cui, Fei, Jiang, Liu, & Wang, 2019 from Mt Leigong of Guizhou; *N. daunchina* (Chang, 1933) from western China; *N. pleuraden* (Boulenger, 1904) from southwestern China; and *N. chapaensis* (Bourret, 1937) and *N. lini* (Chou, 1999) from the northeastern Indochinese peninsula.

Among the species in genus *Nidirana*, *N. adenopleura* has the widest distribution area and has been reported from Taiwan, Fujian, Zhejiang, Anhui, Jiangxi, Guangdong, Guangxi, Hunan and Guizhou (Fei et al. 2009, 2012). In the previous study (Lyu et al. 2017), the populations from Taiwan, northern Fujian, Jingning County of Zhejiang, and Mt Jinggang of Jiangxi were confirmed as the same species, which also synonymized *N. caldwelli* Schmidt, 1925 with *N. adenopleura*. Besides, it is worth noting that the frogs previously considered as *N. adenopleura* from Mt Dayao of Guangxi and Mt Leigong of Guizhou were respectively revealed as two new species, *N. yaoica* and *N. leishanensis*, most recently (Lyu et al. 2019; Li et al. 2019). Nevertheless, the exact taxonomic statuses of other *N. adenopleura* populations from China have not yet be tested.

Through our herpetological surveys throughout southeastern China, we have collected a series of *Nidirana* specimens which were previously reported as *N. adenopleura* (Fei et al. 2009, 2012; Li et al. 2011; Mo et al. 2014). However, comprehensive analyses of molecules, bioacoustics, and morphology have indicated that these specimens are distinctive from all known congeners including the true *N. adenopleura* (designated here as *N. adenopleura* sensu stricto), which suggests they should belong to three unnamed cryptic species. Therefore, based on the results of our present work, we herein describe them as three new species of the genus *Nidirana*.

## Materials and methods

### Taxon sampling

**Figure 1.**
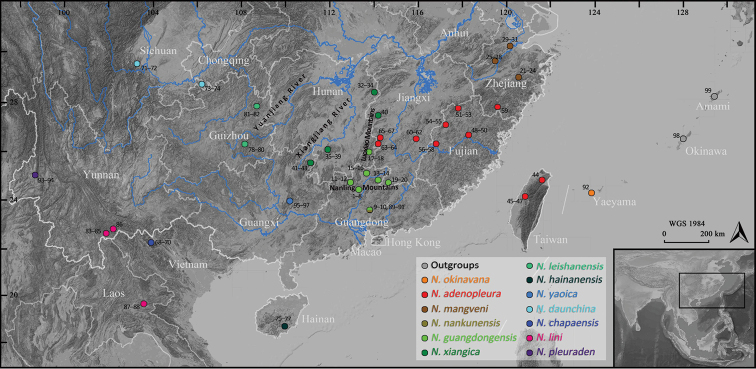
Localities of the *Nidirana* and outgroup *Babina* samples used in this study. Numbers correspond to the ID numbers in Table 1.

For the molecular analysis, a total of 54 muscular samples of *Nidirana* were used, of which 41 are from the undescribed specimens, eight from the true *N. adenopleura*, two from *N. hainanensis* and three from *N. leishanensis*. All samples were attained from euthanatized specimens and then preserved in 95% ethanol and stored at -40 °C. In addition, 43 sequences from all known *Nidirana* congeners and two sequences from the out-group *Babina* Thompson, 1912 (following Lyu et al. 2017) were obtained from GenBank and incorporated into our dataset. Detailed information of these materials is shown in Table 1 and Fig. 1.

### DNA Extraction, PCR amplification, and sequencing

Genomic DNA were extracted from muscle tissue samples, using DNA extraction kit from Tiangen Biotech (Beijing) Co., Ltd. Two mitochondrion genes, namely partial 16S ribosomal RNA gene (16S) and partial cytochrome C oxidase 1 gene (CO1), were amplified. Primers used for 16S were L3975 (5’-CGCCTGTTTACCAAAAACAT-3’) and H4551 (5’-CCGGTCTGAACTCAGATCACGT-3’), and L2A (5’-CCAAACGAGCCTAGTGATAGCTGGTT-3’) and H10 (5’-TGATTACGCTACCTTTGCACGGT-3’), and for CO1 were dgLCO (5’-GGTCAACAAATCATAAAGAYATYGG-3’) and dgHCO (5’-AAACTTCAGGGTGACCAAARAAYCA-3’), following Lyu et al. (2019). PCR amplifications were processed with the cycling conditions that initial denaturing step at 95°C for 4 min, 35 cycles of denaturing at 94°C for 40 s, annealing at 53°C (for 16S) / 48 °C (for CO1) for 40 s and extending at 72°C for 60 s, and a final extending step at 72°C for 10 min. PCR products were purified with spin columns and then sequenced with both forward and reverse primers using BigDye Terminator Cycle Sequencing Kit per the guidelines, on an ABI Prism 3730 automated DNA sequencer by Shanghai Majorbio Bio-pharm Technology Co, Ltd. All sequences were deposited in GenBank (Table 1).

**Table 1.** Localities, voucher information, and GenBank numbers for all samples used in this study. An asterisk denotes type localities.

ID	Species	Localities	Voucher number	16S	CO1
**1**	*Nidirana guangdongensis*	China: Guangdong: Shimentai Nature Reserve *	SYS a005765	MN946404	MN945160
**2**	*Nidirana guangdongensis*	China: Guangdong: Shimentai Nature Reserve *	SYS a005766	MN946405	MN945161
**3**	*Nidirana guangdongensis*	China: Guangdong: Shimentai Nature Reserve *	SYS a005767	MN946406	MN945162
**4**	*Nidirana guangdongensis*	China: Guangdong: Shimentai Nature Reserve *	SYS a005768	MN946407	MN945163
**5**	*Nidirana guangdongensis*	China: Guangdong: Shimentai Nature Reserve *	SYS a005995	MN946408	MN945164
**6**	*Nidirana guangdongensis*	China: Guangdong: Shimentai Nature Reserve *	SYS a005996	MN946409	MN945165
**7**	*Nidirana guangdongensis*	China: Guangdong: Shimentai Nature Reserve *	SYS a005997	MN946410	MN945166
**8**	*Nidirana guangdongensis*	China: Guangdong: Shimentai Nature Reserve *	SYS a005998	MN946411	MN945167
**9**	*Nidirana guangdongensis*	China: Guangdong: Mt Nankun	SYS a005720	MN946412	MN945168
**10**	*Nidirana guangdongensis*	China: Guangdong: Mt Nankun	SYS a005721	MN946413	MN945169
**11**	*Nidirana guangdongensis*	China: Guangdong: Mt Tianjing	SYS a006934	MN946414	MN945170
**12**	*Nidirana guangdongensis*	China: Guangdong: Mt Tianjing	SYS a006935	MN946415	MN945171
**13**	*Nidirana guangdongensis*	China: Guangdong: Mt Chebaling	SYS a007900	MN946416	MN945172
**14**	*Nidirana guangdongensis*	China: Guangdong: Mt Chebaling	SYS a007901	MN946417	MN945173
**15**	*Nidirana guangdongensis*	China: Guangdong: Renhua County	SYS a008135	MN946418	MN945174
**16**	*Nidirana guangdongensis*	China: Guangdong: Renhua County	SYS a008136	MN946419	MN945175
**17**	*Nidirana guangdongensis*	China: Hunan: Mt Bamian	SYS a006195	MN946420	MN945176
**18**	*Nidirana guangdongensis*	China: Hunan: Mt Bamian	SYS a006196	MN946421	MN945177
**19**	*Nidirana guangdongensis*	China: Jiangxi: Mt Jiulian	SYS a004071	MN946422	MN945178
**20**	*Nidirana guangdongensis*	China: Jiangxi: Mt Jiulian	SYS a004082	MN946423	MN945179
**21**	*Nidirana mangveni*	China: Zhejiang: Mt Dapan *	SYS a006310	MN946424	MN945180
**22**	*Nidirana mangveni*	China: Zhejiang: Mt Dapan *	SYS a006311	MN946425	MN945181
**23**	*Nidirana mangveni*	China: Zhejiang: Mt Dapan *	SYS a006312	MN946426	MN945182
**24**	*Nidirana mangveni*	China: Zhejiang: Mt Dapan *	SYS a006313	MN946427	MN945183
**25**	*Nidirana mangveni*	China: Zhejiang: Mt Longmen	SYS a006413	MN946428	MN945184
**26**	*Nidirana mangveni*	China: Zhejiang: Mt Longmen	SYS a006414	MN946429	MN945185
**27**	*Nidirana mangveni*	China: Zhejiang: Mt Longmen	SYS a006415	MN946430	MN945186
**28**	*Nidirana mangveni*	China: Zhejiang: Mt Longmen	SYS a006416	MN946431	MN945187
**29**	*Nidirana mangveni*	China: Zhejiang: Hangzhou City	SYS a007990	MN946432	MN945188
**30**	*Nidirana mangveni*	China: Zhejiang: Hangzhou City	SYNU12050567	KF020600	KF020615
**31**	*Nidirana mangveni*	China: Zhejiang: Hangzhou City	SYNU12050568	KF020601	KF020616
**32**	*Nidirana xiangica*	China: Hunan: Mt Dawei *	SYS a006491	MN946433	MN945189
**33**	*Nidirana xiangica*	China: Hunan: Mt Dawei *	SYS a006492	MN946434	MN945190
**34**	*Nidirana xiangica*	China: Hunan: Mt Dawei *	SYS a006493	MN946435	MN945191
**35**	*Nidirana xiangica*	China: Hunan: Mt Yangming	SYS a007269	MN946436	MN945192
**36**	*Nidirana xiangica*	China: Hunan: Mt Yangming	SYS a007270	MN946437	MN945193
**37**	*Nidirana xiangica*	China: Hunan: Mt Yangming	SYS a007271	MN946438	MN945194
**38**	*Nidirana xiangica*	China: Hunan: Mt Yangming	SYS a007272	MN946439	MN945195
**39**	*Nidirana xiangica*	China: Hunan: Mt Yangming	SYS a007273	MN946440	MN945196
**40**	*Nidirana xiangica*	China: Jiangxi: Mt Wugong	SYS a002590	MN946441	MN945197
**41**	*Nidirana xiangica*	China: Guangxi: Mt Dupangling	SYS a006568	MN946442	MN945198
**42**	*Nidirana xiangica*	China: Guangxi: Mt Dupangling	SYS a006569	MN946443	MN945199
**43**	*Nidirana xiangica*	China: Guangxi: Mt Dupangling	SYS a006570	MN946444	MN945200
**44**	*Nidirana adenopleura*	China: Taiwan: New Taipei City	UMMZ 189963	DQ283117	/
**45**	*Nidirana adenopleura*	China: Taiwan: Taichung City	SYS a007358	MN946445	MN945201
**46**	*Nidirana adenopleura*	China: Taiwan: Taichung City	SYS a007359	MN946446	MN945202
**47**	*Nidirana adenopleura*	China: Taiwan: Taichung City	SYS a007360	MN946447	MN945203
**48**	*Nidirana adenopleura*	China: Fujian: Nanping City	SYS a005911	MF807844	MF807883
**49**	*Nidirana adenopleura*	China: Fujian: Nanping City	SYS a005912	MF807845	MF807884
**50**	*Nidirana adenopleura*	China: Fujian: Nanping City	SYS a005913	MF807846	MF807885
**51**	*Nidirana adenopleura*	China: Fujian: Mt Wuyi	SYS a005939	MF807850	MF807889
**52**	*Nidirana adenopleura*	China: Fujian: Mt Wuyi	SYS a005940	MF807851	MF807890
**53**	*Nidirana adenopleura*	China: Fujian: Mt Wuyi	SYS a005941	MF807852	MF807891
**54**	*Nidirana adenopleura*	China: Fujian: Jiangshi Nature Reserve	SYS a004112	MF807833	MF807872
**55**	*Nidirana adenopleura*	China: Fujian: Jiangshi Nature Reserve	SYS a004132	MF807834	MF807873
**56**	*Nidirana adenopleura*	China: Fujian: Mt Yashu	SYS a005891	MF807841	MF807880
**57**	*Nidirana adenopleura*	China: Fujian: Mt Yashu	SYS a005901	MF807842	MF807881
**58**	*Nidirana adenopleura*	China: Fujian: Mt Yashu	SYS a005902	MF807843	MF807882
**59**	*Nidirana adenopleura*	China: Zhejiang: Jingning County	SYS a002725	MF807827	MF807866
**60**	*Nidirana adenopleura*	China: Jiangxi: Ningdu County	SYS a007089	MN946448	MN945204
**61**	*Nidirana adenopleura*	China: Jiangxi: Ningdu County	SYS a007090	MN946449	MN945205
**62**	*Nidirana adenopleura*	China: Jiangxi: Ningdu County	SYS a007091	MN946450	MN945206
**63**	*Nidirana adenopleura*	China: Jiangxi: Shuichuang County	SYS a004450	MN946456	MN945212
**64**	*Nidirana adenopleura*	China: Jiangxi: Shuichuang County	SYS a004451	MN946457	MN945213
**65**	*Nidirana adenopleura*	China: Jiangxi: Jinggangshan Nature Reserve	SYS a004025	MF807830	MF807869
**66**	*Nidirana adenopleura*	China: Jiangxi: Jinggangshan Nature Reserve	SYS a004026	MF807831	MF807870
**67**	*Nidirana adenopleura*	China: Jiangxi: Jinggangshan Nature Reserve	SYS a004027	MF807832	MF807871
**68**	*Nidirana chapaensis*	Vietnam: Lao Cai: Sapa *	T2483/2000.4850	KR827711	KR087625
**69**	*Nidirana chapaensis*	Vietnam: Lao Cai: Sapa *	1999.5871	KR827710	/
**70**	*Nidirana chapaensis*	Vietnam: Lao Cai: Sapa *	ROM 28070	AF206460	/
**71**	*Nidirana daunchina*	China: Sichuan: Mt Emei *	SYS a004594	MF807822	MF807861
**72**	*Nidirana daunchina*	China: Sichuan: Mt Emei *	SYS a004595	MF807823	MF807862
**73**	*Nidirana daunchina*	China: Sichuan: Hejiang County	SYS a004930	MF807824	MF807863
**74**	*Nidirana daunchina*	China: Sichuan: Hejiang County	SYS a004931	MF807825	MF807864
**75**	*Nidirana hainanensis*	China: Hainan: Mt Diaoluo	SYS a003741	MF807821	MF807860
**76**	*Nidirana hainanensis*	China: Hainan: Mt Diaoluo	SYS a007669	MN946451	MN945207
**77**	*Nidirana hainanensis*	China: Hainan: Mt Diaoluo	SYS a007670	MN946452	MN945208
**78**	*Nidirana leishanensis*	China: Guizhou: Mt Leigong *	CIBLS20150627003	MK293810	MK293828
**79**	*Nidirana leishanensis*	China: Guizhou: Mt Leigong *	CIBLS20150628002	MK293812	MK293830
**80**	*Nidirana leishanensis*	China: Guizhou: Mt Leigong *	SYS a007908	MN946453	MN945209
**81**	*Nidirana leishanensis*	China: Guizhou: Mt Fanjing	SYS a007195	MN946454	MN945210
**82**	*Nidirana leishanensis*	China: Guizhou: Mt Fanjing	SYS a007196	MN946455	MN945211
**83**	*Nidirana lini*	China: Yunnan: Jiangcheng County *	SYS a003967	MF807818	MF807857
**84**	*Nidirana lini*	China: Yunnan: Jiangcheng County *	SYS a003968	MF807819	MF807858
**85**	*Nidirana lini*	China: Yunnan: Jiangcheng County *	SYS a003969	MF807820	MF807859
**86**	*Nidirana lini*	China: Yunnan: Lyuchun County	HNNULC001	KF185066	/
**87**	*Nidirana lini*	Laos: Xieng Khouang	FMNH256531	KR264073	/
**88**	*Nidirana lini*	Laos: Xieng Khouang	FMNH256532	KR264074	/
**89**	*Nidirana nankunensis*	China: Guangdong: Mt Nankun *	SYS a005717	MF807838	MF807877
**90**	*Nidirana nankunensis*	China: Guangdong: Mt Nankun *	SYS a005718	MF807839	MF807878
**91**	*Nidirana nankunensis*	China: Guangdong: Mt Nankun *	SYS a005719	MF807840	MF807879
**92**	*Nidirana okinavana*	Japan: Okinawa: Iriomote Island *	Not given	NC022872	NC022872
**93**	*Nidirana pleuraden*	China: Yunnan: Mt Gaoligong	SYS a003775	MF807816	MF807855
**94**	*Nidirana pleuraden*	China: Yunnan: Mt Gaoligong	SYS a003776	MF807817	MF807856
**95**	*Nidirana yaoica*	China: Guangxi: Mt Dayao *	SYS a007020	MK882276	MK895041
**96**	*Nidirana yaoica*	China: Guangxi: Mt Dayao *	SYS a007021	MK882277	MK895042
**97**	*Nidirana yaoica*	China: Guangxi: Mt Dayao *	SYS a007022	MK882278	MK895043
**98**	*Babina holsti*	Japan: Okinawa *	Not given	NC022870	NC022870
**99**	*Babina subaspera*	Japan: Kagoshima: Amami Island *	Not given	NC022871	NC022871

### Phylogenetic analyses

DNA sequences were aligned by the Clustal W algorithm with default parameters (Thompson et al. 1997) and trimmed with the gaps partially deleted in MEGA 6 (Tamura et al. 2013). Two gene segments, 644 base pairs (bp) of CO1 and 1049 bp of16S, were concatenated seriatim into a 1693-bp sequence, and were further tested in jmodeltest v2.1.2 with Akaike and Bayesian information criteria, all resulting the best-fitting nucleotide substitution models of GTR+I+G. Sequenced data was analyzed using Bayesian inference (BI) in MrBayes 3.2.4 (Ronquist et al. 2012), and maximum likelihood (ML) in RaxmlGUI 1.3 (Silvestro and Michalak 2012). Two independent runs were conducted in a BI analysis, each of which was performed for 10,000,000 generations and sampled every 1000 generations with the first 25% samples were discarded as burn-in, resulting a potential scale reduction factor (PSRF) of < 0.005. In ML analysis, the bootstrap consensus tree inferred from 1000 replicates was used to represent the evolutionary history of the taxa analyzed. Mean genetic distances between and within species were calculated in MEGA 6 using the uncorrected *p*-distance model.

### Bioacoustics analysis

Advertisement calls were recorded in the field at the air temperature of 18–20 °C using a SONY PCM D100 digital sound recorder. The sound files in wave format were sampled at 44.1 kHz with 24 bits in depth. Praat 6.0.27 (Boersma 2001) was used to obtain the oscillogram, sonogram, and power spectrum (window length = 0.005 s). Raven pro 1.5 (Cornell Lab of Ornithology, 2003-2014) was used to quantify the acoustic properties (window size = 1024 points, fast Fourier transform, Hamming window with no overlap). The following measurements were taken for each call: call duration (the time between onset of the first note and offset of the last note in a call), note duration (the time between onset and offset of a note), note rise time (the time between onset and max amplitude of a note), and note interval (the time between adjacent notes in a call). Comparison bioacoustics descriptions of known congeners were obtained from the literature (Matsui and Utsunomiya 1983; Chou 1999; Fei et al. 2007, 2009; Chuaynkern et al. 2010; Lyu et al. 2017, 2019; Li et al. 2019).

### Morphology

Comparison characters of all known congeners were obtained from the literature (Boettger 1895; Boulenger 1904, 1909; Schmidt 1925; Chang and Hsu 1932; Bourret 1937; Kuramoto 1985; Chou 1999; Fei et al. 2007, 2009; Matsui 2007; Chuaynkern et al. 2010; Lyu et al. 2017, 2019; Li et al. 2019) and 74 examined museum specimens of seven species which are listed in the Appendix. All specimens were fixed in 10% buffered formalin and later transferred to 70% ethanol, and deposited in the Museum of Biology, Sun Yat-sen University (**SYS**), Institute of Herpetology, Shenyang Normal University (**SYNU**), Natural History Museum of Guangxi (**NHMG**), and Chengdu Institute of Biology, Chinese Academy of Sciences (**CIB**), China.

Morphological descriptions mainly follow Fei et al. (2009), Chuaynkern et al. (2010) and Lyu et al. (2017). Sex and age were determined by secondary sexual characters, i.e., the presence of suprabrachial glands in males. Webbing formula was written according to Savage (1975). External measurements were made for the unnamed *Nidirana* specimens and 18 specimens of *N. adenopleura*, with digital calipers (Neiko 01407A Stainless Steel 6-Inch Digital Caliper, USA) to the nearest 0.1 mm. These measurements were as follows:

**SVL** snout-vent length (from tip of snout to posterior margin of vent);

**HDL** head length (from tip of snout to the articulation of the jaw);

**HDW** head width (head width at the commissure of the jaws);

**SNT** snout length (from tip of snout to the anterior corner of the eye);

**IND** internasal distance (distance between nares);

**IOD** interorbital distance (minimum distance between upper eyelids);

**ED** eye diameter (from the anterior corner of the eye to posterior corner of the eye);

**TD** tympanum diameter (horizontal diameter of tympanum);

**TED** tympanum-eye distance (from anterior edge of tympanum to posterior corner of the eye);

**HND** hand length (from the proximal border of the outer palmar tubercle to the tip of digit III);

**RAD** radio-ulna length (from the flexed elbow to the proximal border of the outer palmar tubercle);

**FTL** foot length (from distal end of shank to the tip of digit IV);

**TIB** tibial length (from the outer surface of the flexed knee to the heel).

Principal component analysis (PCA), one-way analysis of variance (ANOVA) and Tukey test for multiple comparisons, were performed on the adult male specimens, of which the morphometric measurements were ln-transformed in order to normalize the variables, to test the significance of differences on morphometric characters among different species, using R 3.3.2 (R Core Team 2016).

## Results

### Phylogenetic analyses

**Figure 2.**
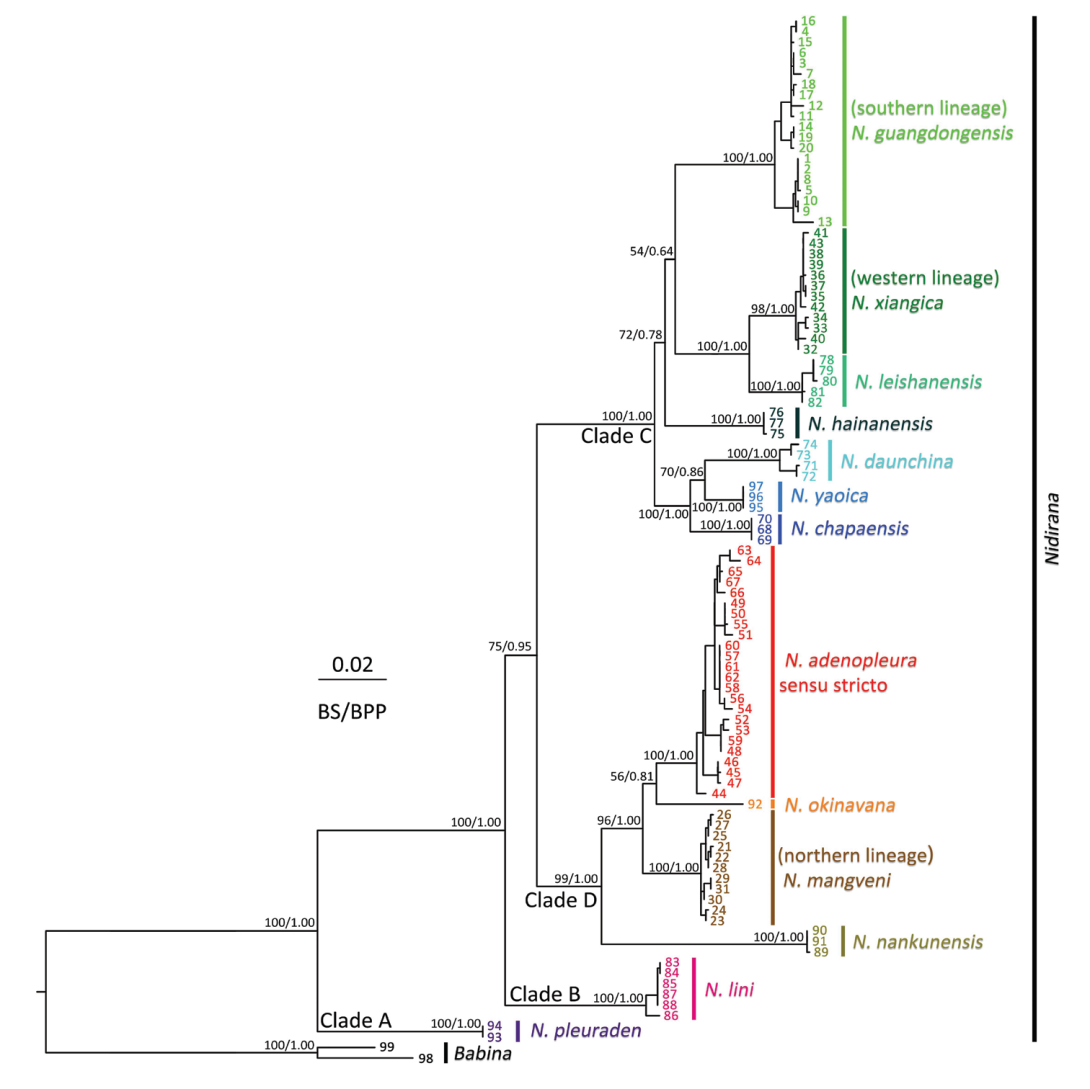
Phylogenetic tree based on mitochondrial 16S + CO1 genes. Numbers at tips of branches correspond to the ID numbers in Table 1.

The ML and BI analyses resulted in essentially identical topologies and were integrated in Fig. 2, in which the major nodes were sufficiently supported with the bootstrap supports (BS) for maximum likelihood analysis > 75 and the Bayesian posterior probabilities (BPP) > 0.95. Mean *p*-distance among all in-group and out-group species used in this study are given in Table 2.

**Table 2.** Mean *p*-distance gene among the *Nidirana* and *Babina* species used in this study.

**ID**	**Species**	**1**	**2**	**3**	**4**	**5**	**6**	**7**	**8**	**9**	**10**	**11**	**12**	**13**	**14**	**15**
1	*Nidirana guangdongensis*	0.6														
2	*Nidirana mangveni*	7.1	0.3													
3	*Nidirana xiangica*	5.0	7.5	0.3												
4	*Nidirana adenopleura*	6.7	3.1	7.6	0.8											
5	*Nidirana chapaensis*	4.4	5.5	4.0	5.2	0.0										
6	*Nidirana daunchina*	5.0	7.1	5.1	6.7	3.0	0.6									
7	*Nidirana hainanensis*	4.6	6.9	4.5	6.8	3.7	5.1	0.0								
8	*Nidirana leishanensis*	5.1	7.3	2.4	6.9	4.4	5.2	4.4	0.2							
9	*Nidirana lini*	6.8	5.6	7.0	6.0	5.0	6.7	6.0	6.4	0.2						
10	*Nidirana nankunensis*	8.4	5.8	8.7	6.3	8.2	9.0	8.5	8.1	7.6	0.0					
11	*Nidirana okinavana*	7.2	3.4	8.2	3.4	5.5	7.3	7.3	7.6	6.6	6.1	/				
12	*Nidirana pleuraden*	9.9	8.5	10.2	8.9	7.8	9.2	9.4	10.4	7.8	10.3	9.3	0.0			
13	*Nidirana yaoica*	4.6	6.7	4.6	6.0	2.4	2.8	4.1	4.5	6.4	8.5	6.8	9.3	0.0		
14	*Babina holsti*	15.0	13.9	15.6	14.3	13.5	14.6	15.0	15.7	13.1	15.1	14.6	12.7	15.0	/	
15	*Babina subaspera*	14.9	13.8	15.4	14.2	13.0	14.5	14.8	15.2	12.9	14.7	14.6	12.7	15.0	3.3	/

In the phylogenetic result, all samples of genus *Nidirana* formed a monophyletic group, which can be further divided into four highly supported clades A, B, C, and D (the names of clades follow Lyu et al. (2017)). This result is consistent with the phylogenic relationship in previous studies (Lyu et al. 2017, 2019; Li et al. 2019). However, the relationship among clades B, C, and D remains unresolved due to the insignificant supported values among these clades.

Within clade D, the samples from Taiwan, northern Fujian, southern Zhejiang and central Jiangxi are grouped in a distinct and substantial single lineage (red color in Figs 1 and 2) that represent the *Nidirana adenopleura* s. s. whose type locality is in Taiwan Island. Nevertheless, the samples from northern Zhejiang (brown color in Figs 1 and 2), which were previously recorded as *N. adenopleura*, are grouped in a distinct single lineage (designated here as the northern lineage), that is non-monophyletic with the lineage of *N. adenopleura* s. s. and has significant divergences against all congeners. In addition, the samples from Xiangjiang River Basin (dark green color in Figs 1 and 2) and from Nanling Mountains and southern Luoxiao Mountains (bright green color in Figs 1 and 2), which were also previously considered as *N. adenopleura*, form two distinct lineages with significant divergences respectively (designated here as the western and southern lineages respectively) within clade C, which are both significantly distant from the lineage of the *N. adenopleura* s. s. in clade D.

This phylogenetic result indicates that the previous identifications for the populations from northern Zhejiang (northern lineage), from Xiangjiang River Basin (western lineage), and from Nanling Mountains and southern Luoxiao Mountains (southern lineage) are incorrect, and these three populations represent three separate evolutionary lineages within the genus *Nidirana*.

### Bioacoustics analysis

**Figure 3.**
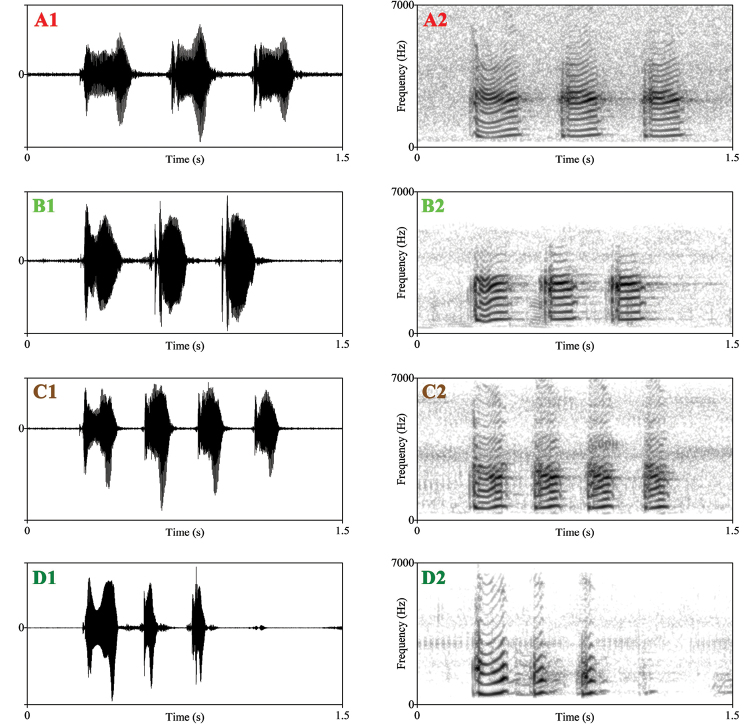
Advertisement call spectrograms. (**A**) *Nidirana adenopleura*; (**B**) *N. guangdongensis* sp. nov.; (**C**) *N. mangveni* sp. nov.; (**D**) *N. xiangica* sp. nov.; (**1**) sonogram; (**2**) waveform.

The call spectrograms of *Nidirana adenopleura* s. s. and the three unnamed lineages are shown in Fig. 3 and the measurement parameters are listed in Table 3.

**Table 3.** Vocalization parameters of *Nidirana adenopleura*, *N. guangdongensis* sp. nov., *N. mangveni* sp. nov., and *N. xiangica* sp. nov.

		***N. adenopleura***	***N. guangdongensis***	***N. mangveni***	***N. xiangica***
Call	Notes number	2–5 (3.4 ± 0.9, *N* = 83)	2–4 (2.9 ± 0.7, *N* = 54)	2–7 (4.6 ± 1.2, *N* = 108)	2–3 (2.8 ± 0.4, *N* = 57)
	Call duration (ms)	525.0–1585.5 (1005.1 ± 341.3, *N* = 83)	445.0–1198.1 (744.6 ± 206.8, *N* = 54)	423.6–1722.7 (967.2 ± 278.9, *N* = 108)	331.9–624.8 (504.3 ± 95.0, *N* = 57)
	Note duration (ms)	153.6–292.4 (212.3 ± 33.0, *N* = 260)	134.0–226.7 (164.3 ± 16.2, *N* = 150)	89.0–203.0 (136.9 ± 23.2, *N* = 462)	/
	Note rise time (ms)	1.4–228.3 (106.1 ± 70.7, *N* = 260)	0.0–138.5 (28.7 ± 32.4, *N* = 150)	4.1–148.6 (79.5 ± 26.9, *N* = 462)	/
	Note interval (ms)	104.0–245.2 (159.5 ± 28.4, *N* = 177)	79.9–262.6 (162.1 ± 26.4, *N* = 96)	59.3–192.7 (116.4 ± 20.8, *N* = 354)	85.0–195.6 (125.8 ± 17.8, *N* = 95)
First note	Note duration (ms)	/	/	/	148.0–233.0 (170.4 ± 14.5, *N* = 57)
	Note rise time (ms)	/	/	/	89.8–149.1 (126.2 ± 17.5, *N* = 57)
Non-first notes	Note duration (ms)	/	/	/	60.1–128.0 (74.6 ± 11.8, *N* = 95)
	Note rise time (ms)	/	/	/	2.2–43.0 (27.8 ± 10.2, *N* = 95)

The advertisement calls of the southern lineage is different from the congeners by (1) containing 2–4 (2.9 ± 0.7, *N* = 54) identical regular notes vs. containing 10–25 fast-repeated regular notes in *Nidirana okinavana*; containing 5–7 regular notes in *N. lini*; containing 4–7 regular notes in *N. pleuraden*; containing 2–4 fast-repeated double-notes in *N. hainanensis*; containing a significantly different first note in *N. daunchina* and *N. nankunensis*; containing a single note in *N. leishanensis*; (2) the call notes last 134.0–226.7 ms vs. the call notes last 30–54 ms in *N. yaoica*; (3) the calls of the southern lineage is similar to that of *N. adenopleura* s. s. but can be distinguished by the relative shorter note duration (164.3 ± 16.2 ms vs. 212.3 ± 33.0 ms) and shorter note rise time (28.7 ± 32.4 ms vs. 106.1 ± 70.7 ms).

The advertisement calls of the northern lineage is different from the congeners by (1) containing 2–7 (4.6 ± 1.2, *N* = 108) identical regular notes vs. containing 10–25 fast-repeated regular notes in *Nidirana okinavana*; containing 2–4 fast-repeated double-notes in *N. hainanensis*; containing a significantly different first note in *N. daunchina* and *N. nankunensis*; containing a single note in *N. leishanensis*; (2) the call notes last 89.0–203.0 ms vs. the call notes last 30–54 ms in *N. yaoica*; (3) the calls of the southern lineage is similar to that of *N. adenopleura* s. s. but can be distinguished by the relative shorter note duration (136.9 ± 23.2 ms vs. 212.3 ± 33.0 ms) and shorter note rise time (79.5 ± 26.9 ms vs. 106.1 ± 70.7 ms); (4) the calls of the southern lineage is similar to that of the southern lineage but can be distinguished by more note number in per call (2–7, 4.6 ± 1.2 vs. 2–4, 2.9 ± 0.7).

The advertisement calls of the western lineage is different from the congeners by (1) containing a significantly different first note vs. containing several identical regular notes in *Nidirana adenopleura*, southern lineage, northern lineage, *N. yaoica*, *N. chapaensis*, *N. lini*, and *N. pleuraden*; containing 2–4 fast-repeated double-notes in *N. hainanensis*; containing a single note in *N. leishanensis*; (2) containing 2–3 notes vs. containing 10–25 fast-repeated regular notes in *N. okinavana*; containing 13–15 fast-repeated notes in *N. nankunensis*; (3) the calls of the western lineage is similar to that of *N. daunchina* but can be distinguished by the relative shorter note intervals time (125.8 ± 17.8 ms vs. 193.6 ± 26.3 ms) and shorter duration of non-first notes (74.6 ± 11.8 ms vs. 140.6 ± 5.6 ms).

### Morphology

**Figure 4.**
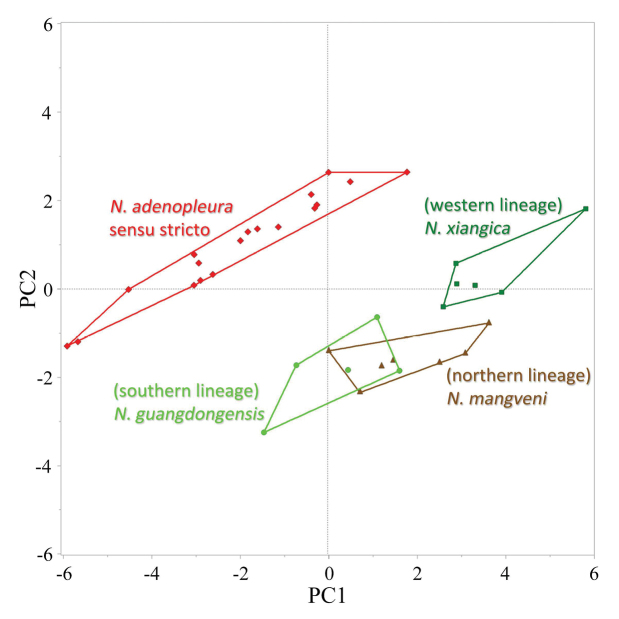
Scatter plot of PC1 and PC2 of Principal Component Analysis based on the morphometric measurements of male specimens of *Nidirana adenopleura*, *N. guangdongensis* sp. nov., *N. mangveni* sp. nov., and *N. xiangica* sp. nov.

The results of PCA based on morphometric measurements of the male specimens of *Nidirana adenopleura* s. s. and the three unnamed lineages are shown in Fig. 4. The extracted components PC1 eigenvectors accounted for 58.8% of the variance, PC2 for 18.6%, PC3 for 6.12%, and PC4 for 5.46%, which cumulate 88.98% of the variance. As shown on the scatter plot of PC1 and PC2, the specimens of *N. adenopleura* s. s., are significantly different from the specimens of the other three lineages. The specimens of the western lineage are also well separated from others. However, the specimens of the southern and northern lineages, which are significantly distant from each other in the phylogenetic tree, overlap with each other in the PCA result.

The results of one-way ANOVA and Tukey test for multiple comparisons are given in Table 4. The results indicate that all morphometric data are significantly different among *Nidirana adenopleura* s. s. and the three unnamed lineages (all *p*-values < 0.05). Specifically, for the specimens of *N. adenopleura* s. s. and the unnamed northern lineage which phylogenetically clustered within clade D together, they are significantly different in SVL, HDL, ED, RAD, FTL, and TIB. For the specimens of the unnamed western and southern lineages which phylogenetically clustered within clade C together, they are significantly different in HDW, SNT, IND, ED, TD, and RAD.

**Table 4.** Morphometric comparisons based on the morphometric measurements of male specimens of *Nidirana adenopleura* (*N* = 18), *N. guangdongensis* sp. nov. (*N* = 5), *N. mangveni* sp. nov. (*N* = 7), and *N. xiangica* sp. nov. (*N* = 6). *p-values < 0.05, **p-values < 0.01, ***p-values < 0.001.

	***p*-value**						
	ANOVA	*adenopleura* vs *guangdongensis*	*adenopleura* vs *mangveni*	*adenopleura* vs *xiangica*	*guangdongensis* vs *mangveni*	*guangdongensis* vs *xiangica*	*mangveni* vs *xiangica*
**SVL**	0.001 **	0.455	0.014 *	0.001 **	0.646	0.201	0.770
**HDL**	0.000 ***	0.886	0.004 **	0.002 **	0.133	0.074	0.980
**HDW**	0.000 ***	0.980	0.053	0.000 ***	0.336	0.005 *	0.164
**SNT**	0.009 *	1.000	0.993	0.006 *	0.997	0.044 *	0.041 *
**IND**	0.001 **	1.000	0.237	0.001 **	0.436	0.007 *	0.141
**IOD**	0.013 *	0.975	0.501	0.008 *	0.890	0.109	0.292
**ED**	0.000 ***	0.068	0.000 ***	0.000 ***	0.028 *	0.012 *	0.963
**TD**	0.001 **	0.999	0.052	0.001 **	0.159	0.011 *	0.533
**TED**	0.005 *	0.784	0.065	0.203	0.656	0.107	0.003 **
**HND**	0.017 *	0.582	0.308	0.013 *	0.995	0.458	0.530
**RAD**	0.000 ***	0.000 ***	0.000 ***	0.000 ***	0.729	0.027 *	0.158
**FTL**	0.000 ***	0.000 ***	0.000 ***	0.000 ***	0.976	0.278	0.092
**TIB**	0.000 ***	0.000 ***	0.000 ***	0.000 ***	0.997	0.371	0.413

Detail comparisons among specimens of the western, southern, and northern lineages and all recognized congeners are listed in Table 5. The populations of the southern, northern, and western lineages can be readily and consistently distinguished from all other species by a combination of characteristics (see Comparisons below).

**Table 5.** Diagnostic characters separating all species of genus *Nidirana*.

**Species**	**SVL of males (mm)**	**SVL of females (mm)**	**Fingers tips**	**Lateroventral groove on fingers**	**Relative length of fingers**	**Toes tips**	**Lateroventral groove on toes**	**Tibio-tarsal articulation**	**Subgular vocal sacs**	**Nuptial pad**
*N. guangdongensis*	50.0–58.4	55.3–59.3	Dilated	Present except finger I	II < I < IV < III	Dilated	Present	Nostril	Present	One on finger I
*N. mangveni*	53.6–59.7	59.7–65.1	Dilated	Present on fingers III and IV	I < II < IV < III	Dilated	Present	Anterior corner of eye	Present	One on finger I
*N. xiangica*	56.3–62.3	53.5–62.6	Dilated	Present	II < I < IV < III	Dilated	Present	Eye-snout	Present	One on finger I
*N. adenopleura*	43.1–57.6	47.6–60.7	Dilated	Present except finger I	II < I < IV < III	Dilated	Present	Snout tip or eye-snout	Present	One on finger I
*N. nankunensis*	33.3–37.1	37.8–39.5	Dilated	Present except finger I	II < I < IV < III	Dilated	Present	Nostril	Present	One on finger I
*N. okinavana*	35.5–42.8	44.6–48.8	Dilated	Present except finger I	II < I < IV < III	Dilated	Present	Eye center-near nostril	Absent	Poorly one on finger I
*N. daunchina*	40.6–51.0	44.0–53.0	Dilated	Absent or rarely present	II < I < IV < III	Dilated	Present	Nostril	Present	One on finger I
*N. yaoica*	40.4–45.9	?	Dilated	Present	II < I < IV < III	Dilated	Present	Nostril	Present	One on finger I
*N. chapaensis*	35.5–42.5	41.0–51.8	Dilated	Present except finger I	II < I = IV < III	Dilated	Present	Nostril	Present	Two on finger I
*N. hainanensis*	32.8–44.4	?	Dilated	Present	II < I < IV < III	Dilated	Present	Nostril	Present	Absent
*N. leishanensis*	49.5–56.4	43.7–55.3	Dilated	Present	II < IV < I < III	Dilated	Present	Eye-snout	Present	Two on fingers I and II
*N. lini*	44.1–63.1	57.7–68.6	Dilated	Present except finger I	II < I < IV < III	Dilated	Present	Beyond snout	Present	One on finger I
*N. pleuraden*	45.4–58.7	45.5–62.5	Not dilated	Absent	II < I < IV < III	Not dilated	Absent	Eye-snout	Present	One on finger I

**Table 5. Continued.** 

Species	Spinules on dorsal skin	Nest construction	Tadpole labial tooth row formula	Calling	Cites
*N. guangdongensis*	Entire	Absent	?	2–4 regular notes	This study
*N. mangveni*	Entire or posterior	Absent	?	2–7 regular notes	This study
*N. xiangica*	Entire	Absent	?	2–3 notes containing a specific first note	This study
*N. adenopleura*	Entire or posterior	Absent	1:1+1/1+1:2 or 1:0+0/1+1:1	2–5 regular notes	Pope (1931); Chuaynkern et al. (2010); Lyu et al. (2017); this study
*N. nankunensis*	Absent or few above vent	Present	1:1+1/1+1:2	13–15 fast-repeated notes containing a specific first note	Lyu et al. (2017)
*N. okinavana*	Absent	Present	1:1+1/1+1:2	10–25 fast-repeated notes	Matsui and Utsunomiya (1983); Chuaynkern et al. (2010)
*N. daunchina*	Absent	Present	1:1+1/1+1:2 or 1:1+1/2+2:1	2–5 notes containing a specific first note	Liu (1950); Fei et al. (2009); Lyu et al. (2017)
*N. yaoica*	Absent	?	?	1–3 fast-repeated regular notes	Lyu et al. (2019)
*N. chapaensis*	Absent or few above vent	Present	1:1+2/1+1:2	3 notes	Chuaynkern et al. (2010)
*N. hainanensis*	Absent	Present	?	2–4 fast-repeated double-notes	Fei et al. (2009, 2012); Lyu et al. (2017)
*N. leishanensis*	Absent	Absent	1:1+2/ 1+1:2	1 single note	Li et al. (2019)
*N. lini*	Posterior	Absent	1:1+1/1+1:2	5–7 notes	Chou (1999); Fei et al. (2009); Lyu et al. (2017)
*N. pleuraden*	Posterior	Absent	1:1+1/1+1:2 or 1:1+1/2+2:1	4–7 notes	Fei et al. (2009); Lyu et al. (2017)

### Conclusion

Based on the results of molecular, bioacoustic, and morphological analyses, the populations of the southern, northern and western lineages are significantly different from all congeners of genus *Nidirana*, including the *N. adenopleura* s. s. Thus, we propose these three linages as three new species, i.e., *Nidirana guangdongensis* sp. nov. for the population from Nanling Mountains and southern Luoxiao Mountains (southern lineage), *Nidirana mangveni* sp. nov. for the population from northern Zhejiang (northern lineage), and *Nidirana xiangica* sp. nov. for the population from Xiangjiang River Basin (western lineage).

### Taxonomic accounts

#### Nidirana guangdongensis

Taxon classificationAnimaliaAnuraRanidae

Lyu, Wan, & YY Wang sp. nov.

2F2B32F6-F797-5F1E-AFF6-5EFD6B51D9F4

http://zoobank.org/52CE0A4A-BDC1-4E5B-B2C3-7A58FDABE24F

Figures 5 , 6 , 7

##### Chresonymy.

*Nidirana adenopleura*: Fei et al. 2009, 2012; Li et al. 2011

##### Holotype.

**Figure 5.**
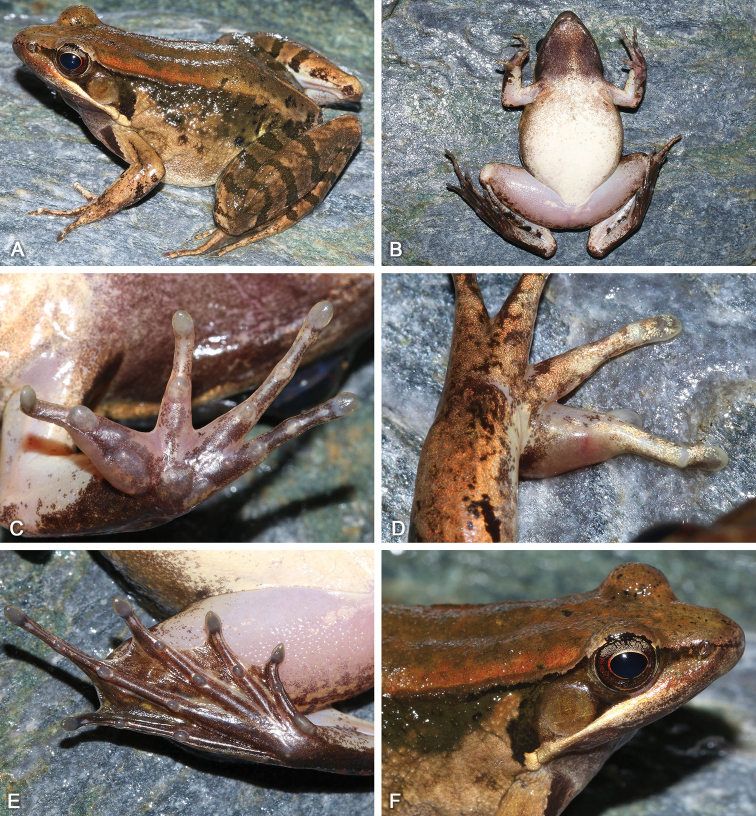
Morphological features of the adult male holotype SYS a005767 of *Nidirana guangdongensis* sp. nov. in life. (**A**) dorsolateral view; (**B**) ventral view; (**C**) left hand; (**D**) nuptial pad; (**E**) right foot; (**F**) close-up of head showing the dense white horny spinules on dorsum, upper eyelid, while absent on temporal regions.

**Figure 6.**
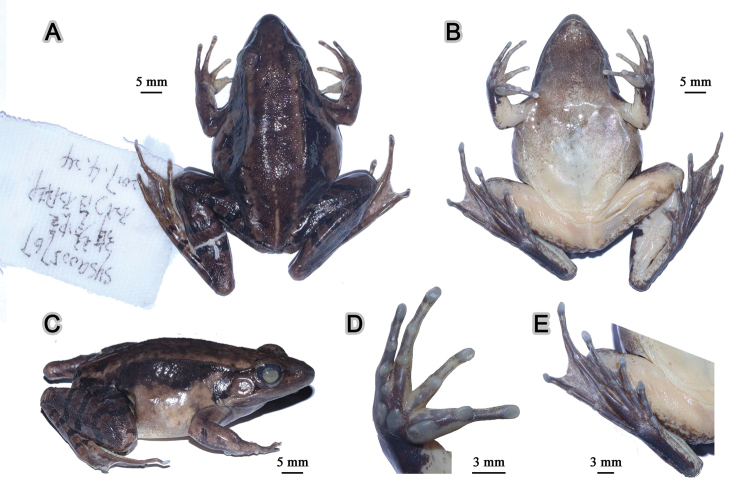
Morphological features of the adult male holotype SYS a005767 of *Nidirana guangdongensis* sp. nov. in preservative. (**A**) dorsal view; (**B**) ventral view; (**C**) lateral view; (**D**) right hand; (**E**) right foot.

SYS a005767 (Figs 5, 6), adult male, collected by Zhi-Tong Lyu on 24 April 2017 from Shimentai Nature Reserve (24.4450°N, 113.1617°E; ca. 320 m a.s.l.), Yingde City, Guangdong Province, China.

##### Paratypes.

Seven adult specimens from the same locality as the holotype. Male SYS a005765 and female SYS a005766, collected by Zhi-Tong Lyu and Yuan-Qiu Li at the same time as the holotype; male SYS a005995 and females SYS a005997–98, collected by Zhi-Tong Lyu, Yong-You Zhao and Chao-Yu Lin on 20 June 2017; male SYS a006879/CIB 107273 collected by Zhi-Tong Lyu, Yong-You Zhao and Yuan-Qiu Li on 20 April 2018; male SYS a007688 collected by Yu-Long Li, Can-Zhong Rong and Yuan-Qiu Li on 23 April 2019.

##### Etymology.

The species name *guangdongensis* refers to Guangdong (广东), also known as Yue (粤), which is the province where the type locality, Shimentai Nature Reserve, belongs to.

##### Differential diagnosis.

*Nidirana guangdongensis* sp. nov. is distinguished from its congeners by the following combination of the morphological characteristics: (1) body large and elongated, with SVL 50.0–58.4 (53.9 ± 3.3, *N* = 5) mm in adult males, and SVL 55.3–59.3 (57.0 ± 2.1, *N* = 3) mm in adult females; (2) disks of digits dilated, rounded; (3) lateroventral grooves present on every digit except finger I; (4) heels overlapping; (5) tibio-tarsal articulation reaching the nostril; (6) mid-dorsal stripe present on posterior dorsum; (7) week supernumerary tubercles below the base of each finger, palmar tubercles prominent and distinct; (8) supratympanic fold absent; (9) white horny spinules on the entirely dorsum, dorsolateral folds, flanks and dorsal hindlimbs, while absent on temporal regions in males; (10) a pair of subgular vocal sacs present; (11) one single nuptial pad present on the finger I, nuptial spinules invisible; (12) suprabrachial gland large and smooth, prominent; (13) calling: 2–4 identical regular notes.

##### Comparison.

Morphologically, *Nidirana guangdongensis* sp. nov. is unique when compared with all known congeners by the combination of the following characteristics: (1) large body size, SVL 50.0–58.4 mm in males and 55.3–59.3 mm in females vs. < 48.0 mm in males or < 53.0 mm in females in *N. nankunensis*, *N. okinavana*, *N. daunchina*, *N. yaoica*, *N. chapaensis* and *N. hainanensis*; (2) relative finger lengths II < I < IV < III vs. II < I = IV < III in *N. chapaensis*; vs. II < IV < I < III in *N. leishanensis*; (3) presence of lateroventral groove on every digit except finger I vs. absent on fingers and toes in *N. pleuraden*; vs. absent or barely visible on fingers in *N. daunchina*; vs. present on finger I in *N. yaoica*, *N. leishanensis* and *N. hainanensis*; (4) tibio-tarsal articulation reaches at the nostril vs. beyond the snout tip in *N. lini*; (5) white horny spinules on the entirely dorsum and flanks in males vs. absent on dorsum and flanks or few above vent in *N. nankunensis*, *N. okinavana*, *N. daunchina*, *N. yaoica*, *N. chapaensis*, *N. leishanensis* and *N. hainanensis*; vs. present on dorsum while absent on flanks in *N. adenopleura*, *N. lini* and *N. pleuraden*; (6) the presence of a single nuptial pad on finger I vs. absent in *N. hainanensis*; vs. divided into two parts in *N. chapaensis*; vs. two nuptial pads on fingers I and II respectively; (7) the presence of a pair of subgular vocal sacs vs. absent in *N. okinavana*.

##### Description of holotype.

SYS a005767 (Figs 5, 6), adult male. Body large and elongated, SVL 55.2 mm; head longer than wide (HDW/HDL 0.90), flat above; snout rounded in dorsal and lateral views, slightly protruding beyond lower jaw, longer than horizontal diameter of eye (SNT/ED 1.30); canthus rostralis distinct, loreal region concave; nostril round, directed laterally, closer to the snout than to the eye; a longitudinal swollen mandibular ridge extending from below nostril through lower edges of eye and tympanum to above insertion of arm, where the ridge is intermittent, forming a maxillary gland and shoulder gland; supratympanic fold absent; interorbital space flat, narrower than internasal distance (IND/IOD 1.24); pupil elliptical, horizontal; tympanum distinct, round, TD/ED 0.86, and close to eye, TED/TD 0.32; pineal ocellus slightly visible; vomerine ridge present, bearing small teeth; tongue large, cordiform, notched behind; a pair of subgular vocal sacs present.

Forelimbs moderately robust, lower arm 0.17 of SVL and hand 0.27 of SVL; fingers thin, relative finger lengths II < I < IV < III; tip of each finger slightly dilated, forming rounded disks; lateroventral grooves on all fingers except finger I, not meeting at the tip of disks; fingers free of webbing; presence of distinct lateral fringes on inner and outer sides of fingers II, III and IV, and on outer side of finger I; subarticular tubercles prominent and rounded; week supernumerary tubercles below the base of each finger; three elliptic, large, prominent and very distinct palmar tubercles; a single nuptial pad on the dorsal surface of first finger, nuptial spinules invisible.

Hindlimbs relatively robust, tibia 0.54 of SVL and foot 0.77 of SVL; heels overlapping when hindlimbs flexed at right angles to axis of body; tibio-tarsal articulation reaching the nostril when hindlimb is stretched along the side of the body; toes relatively long and thin, relative lengths I < II < V < III < IV; tip of each toe slightly dilated with remarkable elongated ventral callous pad, forming long and pointed disk; well-developed lateroventral grooves on toes , not meeting at the tip of disks; webbing moderate, webbing formula: I 1⅓ - 2 II 1⅓ - 2⅓ III 1⅔ - 3 IV 3⅓ - 1⅓ V; presence of lateral fringes on inner and outer sides of each toes, forming distinct dermal flap on the lateral edges of toes I and V; subarticular tubercles rounded, prominent; inner metatarsal tubercle elliptic, length triple the width; outer metatarsal tubercle indistinct, small and rounded; tarsal folds and tarsal tubercle absent.

Dorsal surface rough with dense horny spinules; developed dorsolateral fold with sparse horny spinules from posterior margin of upper eyelid to above groin but intermittent posteriorly; flank rough with dense tubercles and dense horny spinules; a large and smooth suprabrachial gland behind base of forelimb, prominent; dorsal surface of forelimb relatively smooth without horny spinules, weak longitudinal ridges on upper arms and slightly extending to lower arm; the dorsal surfaces of thigh and tibia rough with dese tubercles and dense horny spinules, forming several longitudinal ridges. Ventral surface of throat, body, and limbs smooth; large flattened tubercles densely arranged on the rear of thigh and around vent.

##### Coloration of holotype.

In life (Fig. 5), dorsal surface reddish brown; horny spinules on the skin white; pineal ocellus yellowish; a yellowish mid-dorsal stripe on the posterior dorsum; dorsolateral fold dark brown; upper flank dark brown; lower flank light brown; suprabrachial gland light brown. Dorsal forelimbs light brown; a longitudinal black stripe on the anterior surface of the forelimb; dorsal hindlimbs dark brown, four dark crossbars on the thigh, three on the tibia and three on the tarsus. Loreal and temporal regions dark brown, tympanum light brown; upper ⅓ iris brownish white and lower ⅔ iris reddish brown; maxillary gland and shoulder gland white. Throat dark purplish brown; ventral surface of body and limbs creamy white; rear thigh tinged with pink; ventral hand white with large purplish brown patches; ventral foot purplish brown.

In preservative (Fig. 6), dorsal surface faded with the pineal ocellus and mid-dorsal stripe clearer; white spinules more distinct; dorsal limbs faded with the crossbars more distinct; ventral surface faded, throat grey.

##### Variations.

**Figure 7.**
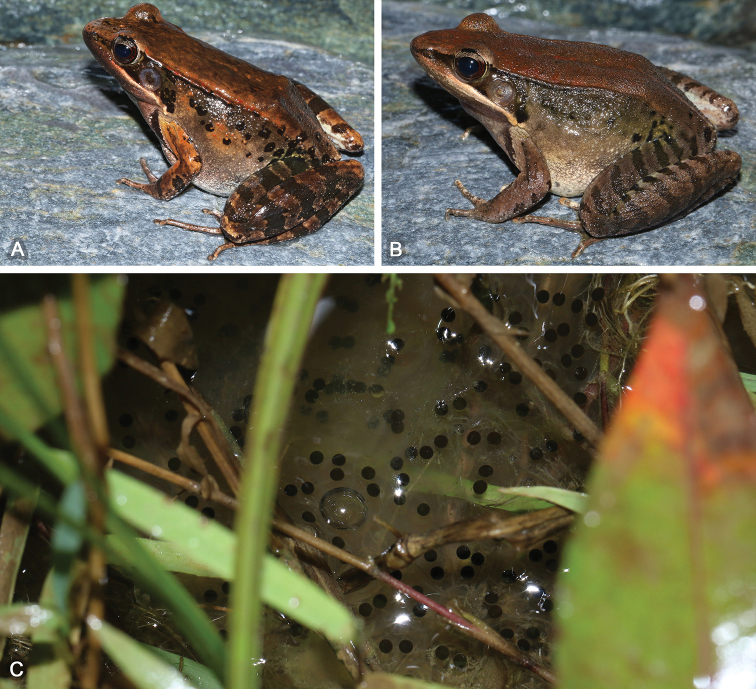
(**A**) adult female paratype SYS a005766 of *Nidirana guangdongensis* sp. nov.; (**B**) adult male paratype SYS a005765; (**C**) eggs in the water surface found in Mt Bamian.

Measurements of type series are given in Table 6. All specimens were similar in morphology. Females (57.0 ± 2.1 mm, *N* = 3) (Fig. 7A) are not significantly larger than males (53.9 ± 3.3 mm, *N* = 5), but relatively smooth than males, not bearing white horny spinules on the dorsum, dorsolateral folds, and flanks. Pineal ocellus invisible in SYS a005765 (Fig. 7B); numerous black spots on flanks in SYS a005766.

**Table 6.** Measurements (in mm) of the type series of *Nidirana guangdongensis* sp. nov. An asterisk denotes the holotype.

	SYS a005767*	**SYS a005765**	**SYS a005995**	**SYS a006879 /CIB 107273**	**SYS a007688**	**SYS a005766**	**SYS a005997**	**SYS a005998**
**Sex**	**Male**	**Male**	**Male**	**Male**	**Male**	**Female**	**Female**	**Female**
SVL	55.2	51.3	50.0	58.4	54.6	56.4	59.3	55.3
HDL	19.4	18.9	18.5	20.8	20.3	20.6	22.2	21.0
HDW	17.5	17.4	17.7	18.5	18.0	18.1	18.6	18.5
SNT	7.9	7.8	7.4	7.9	8.0	8.1	8.6	8.0
IND	5.7	5.8	5.3	5.8	5.6	5.9	6.2	5.5
IOD	4.6	4.7	4.3	4.9	4.9	5.4	5.2	5.1
ED	6.1	5.3	5.6	5.9	5.4	6.1	6.1	5.8
TD	5.2	4.1	3.8	4.9	4.1	4.6	4.2	4.7
TED	1.7	1.5	1.3	1.2	1.3	1.5	1.2	1.4
HND	14.7	13.3	13.8	14.4	14.3	14.4	14.6	15.8
RAD	9.4	8.7	8.6	9.9	8.9	9.0	9.7	9.7
FTL	42.7	39.0	40.1	45.3	43.9	45.5	46.9	47.0
TIB	29.6	27.0	25.4	30.0	29.2	30.1	31.6	31.9

##### Distribution and ecology.

Currently, *Nidirana guangdongensis* sp. nov. is known from northern Guangdong, southern Jiangxi and southeastern Hunan, indicating that this frog is distributed in the Nanling Mountains and southern Luoxiao Mountains of southern China. The frog inhabits in natural ponds. The adult males call at the water surface and the females oviposit directly into the water (Fig. 7C) from April to June. The tadpoles of this species remain unknown. In Mt Nankun, *N. guangdongensis* sp. nov. is sympatric with *N. nankunensis* in the same pond and is more abundant.

##### Vocalization.

The advertisement call (*N* = 54) of *Nidirana guangdongensis* sp. nov. contains 2–4 repeated, identical, regular notes. The two-note call has a duration of 445.0–559.0 (520.6 ± 27.4, *N* = 19) ms; the three-note call has a duration of 681.5–875.8 (794.6 ± 46.4, *N* = 28) ms; the four-note call has a duration of 1117.6–1198.1 (1152.9 ± 29.8, *N* = 7) ms. The notes last 134.0–226.7 (164.3 ± 16.2, *N* = 150) ms with the rise time 0.0–138.5 (28.7 ± 32.4, *N* = 150) ms, and the intervals last 79.9–262.6 (162.1 ± 26.4, *N* = 96) ms.

#### Nidirana mangveni

Taxon classificationAnimaliaAnuraRanidae

Lyu, Qi, & YY Wang sp. nov.

7B7D9018-556D-584F-AF1B-D81B5DEF7276

http://zoobank.org/D4BC572F-FAA8-41A8-856D-3D183FA2AC09

Figures 8 , 9 , 10

##### Chresonymy.

*Nidirana adenopleura*: Fei et al. 2009, 2012

##### Holotype.

**Figure 8.**
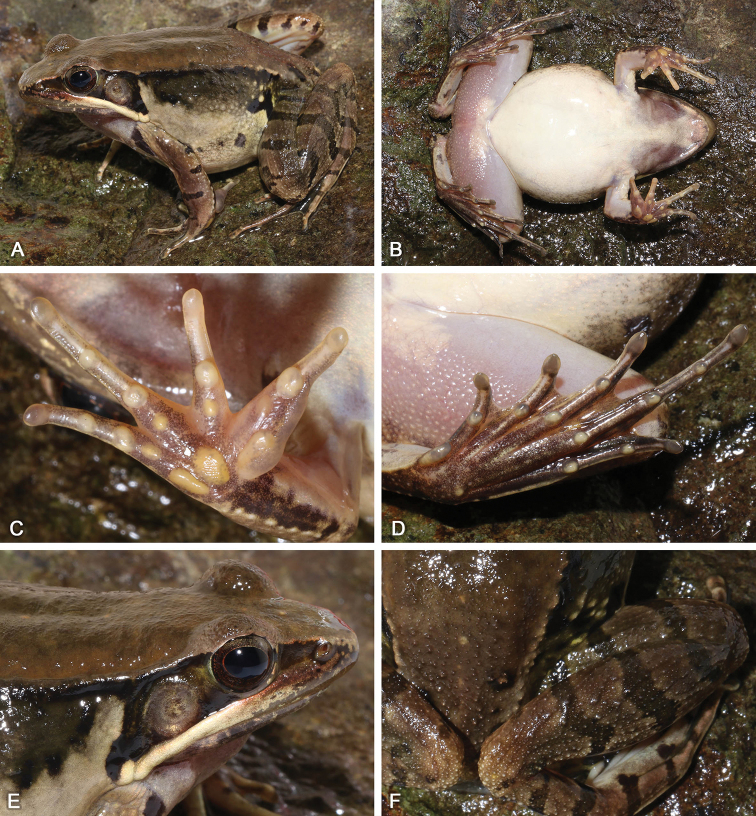
Morphological features of the adult male holotype SYS a006313 of *Nidirana mangveni* sp. nov. in life. (**A**) dorsolateral view; (**B**) ventral view; (**C**) right hand; (**D**) left foot; (**E**) close-up of head showing the week supratympanic fold; (**F**) close-up of posterior dorsum and hindlimb showing the horny spinules.

**Figure 9.**
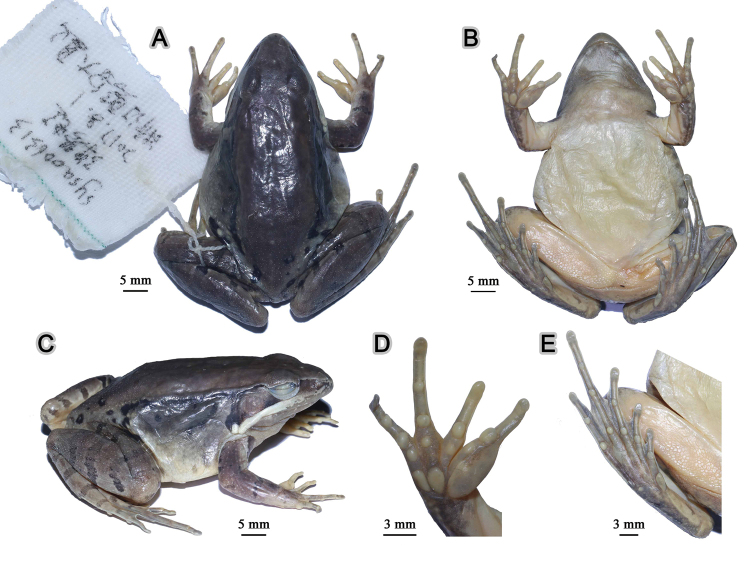
Morphological features of the adult male holotype SYS a006313 of *Nidirana mangveni* sp. nov. in preservative. (**A**) dorsal view; (**B**) ventral view; (**C**) lateral view; (**D**) right hand; (**E**) right foot.

SYS a006313 (Figs 8, 9), adult male, collected by Jian Wang and Zhao-Chi Zeng on 1 August 2017 from Mt Dapan (28.9801°N, 120.5447°E; ca 860 m a.s.l.), Pan’an County, Zhejiang Province, China.

##### Paratypes.

Eight adult specimens. Males SYS a006311–12, SYS a006314/CIB 107275, and female SYS a006310, collected by Jian Wang and Zhao-Chi Zeng at the same time from the same locality as the holotype; males SYS a006413–14 and female SYS a006416, collected by Jian Wang and Zhao-Chi Zeng on 3 August 2017 from Mt Longmen (29.8643°N, 119.9790°E; ca 540m a.s.l.), Fuyang District, Hangzhou City, Zhejiang Province, China; male SYNU 12050569 collected by Zheng-Yan Zhou on 8 May 2012 from Hangzhou Botanical Garden (30.2544°N, 120.1226°E; ca 100m a.s.l.), Xihu District, Hangzhou City, Zhejiang Province, China.

##### Etymology.

The species name *mangveni* refers to Professor Mangven L. Y. Chang (= Meng-Wen Zhang, 张孟闻), an outstanding zoologist born in Ningbo City of northern Zhejiang, who contributed mostly on Chinese herpetological taxonomy and natural history. He is also the author of *Nidirana daunchina*, a congener of this new species.

##### Differential diagnosis.

*Nidirana mangveni* sp. nov. is distinguished from its congeners by the following combination of the morphological characteristics: (1) body large and elongated, with SVL 53.6–59.7 (56.2 ± 2.5, *N* = 7) mm in adult males, and SVL 62.4 ± 3.8 (59.7–65.1, *N* = 2) mm in adult females; (2) disks of digits dilated, rounded; (3) lateroventral grooves present on fingers III and IV, and each toes; (4) relative finger lengths I < II < IV < III; (5) heels overlapping; (6) tibio-tarsal articulation reaching the anterior corner of eye; (7) week supratympanic fold present; (8) mid-dorsal stripe absent or present on posterior dorsum; (9) posterior of dorsal skin rough with dense tubercles; (10) developed supernumerary tubercles below the base of each finger, palmar tubercles prominent and distinct; (11) white horny spinules on the posterior or entire dorsum in males; (12) a pair of subgular vocal sacs present; (13) one single nuptial pad present on the finger I, nuptial spinules invisible; (14) suprabrachial gland large; (15) calling: 2–7 identical regular notes.

##### Comparison.

Morphologically, *Nidirana mangveni* sp. nov. is unique when compared with all recognized congeners by the combination of the following characteristics: (1) large body size, SVL 53.6–59.7 mm in males and 59.7–65.1 mm in females vs. < 53.0 mm in males or females in *N. nankunensis*, *N. okinavana*, *N. daunchina*, *N. yaoica*, *N. chapaensis* and *N. hainanensis*; (2) relative finger lengths I < II < IV < III vs. II < I = IV < III in *N. chapaensis*; vs. II < IV < I < III in *N. leishanensis*; vs. II < I < IV < III in all other congeners; (3) absent of lateroventral groove on fingers I and II vs. absent on fingers and toes in *N. pleuraden*; vs. absent or barely visible on fingers in *N. daunchina*; vs. present on finger II in all other congeners; (4) tibio-tarsal articulation reaches at the anterior corner of eye vs. beyond the snout tip in *N. lini*; vs. at the nostril in *N. guangdongensis*, *N. nankunensis*, *N. daunchina*, *N. yaoica*, *N. chapaensis* and *N. hainanensis*; (5) week supratympanic fold present vs. absent in *N. guangdongensis*, *N. adenopleura*, *N. nankunensis*, *N. daunchina*, *N. yaoica*, *N. hainanensis*, and *N. lini*; (6) white horny spinules on the posterior or entire dorsum in males vs. absent on dorsum or few above vent in *N. nankunensis*, *N. okinavana*, *N. daunchina*, *N. yaoica*, *N. chapaensis*, *N. leishanensis* and *N. hainanensis*; (7) the presence of a single nuptial pad on finger I vs. absent in *N. hainanensis*; vs. divided into two parts in *N. chapaensis*; vs. two nuptial pads on fingers I and II respectively; (8) the presence of a pair of subgular vocal sacs vs. absent in *N. okinavana*.

##### Description of holotype.

SYS a006313 (Figs 8, 9), adult male. Body large and elongated, SVL 54.0 mm; head longer than wide (HDW/HDL 0.87), flat above; snout rounded in dorsal and lateral views, slightly protruding beyond lower jaw, longer than horizontal diameter of eye (SNT/ED 1.22); canthus rostralis distinct, loreal region concave; nostril round, directed laterally, closer to the snout than to the eye; a longitudinal swollen mandibular ridge extending from below nostril through lower edges of eye and tympanum to above insertion of arm, where the ridge is intermittent, forming a maxillary gland and shoulder gland; week supratympanic fold present; interorbital space flat, narrower than internasal distance (IND/IOD 1.25); pupil elliptical, horizontal; tympanum distinct, round, TD/ED 0.73, and close to eye, TED/TD 0.31; pineal ocellus present; vomerine ridge present, bearing small teeth; tongue large, cordiform, notched behind; a pair of subgular vocal sacs present.

Forelimbs moderately robust, lower arm 0.18 of SVL and hand 0.26 of SVL; fingers thin, relative finger lengths I < II < IV < III; tip of each finger slightly dilated, forming rounded disks; lateroventral grooves on fingers III and IV, not meeting at the tip of disks; fingers free of webbing; presence of distinct lateral fringes on inner and outer sides of fingers II, III and IV, absent on finger I; subarticular tubercles prominent and rounded; developed supernumerary tubercles below the base of each finger; three elliptic, large, prominent and very distinct palmar tubercles; a single nuptial pad on the dorsal surface of first finger, nuptial spinules invisible.

Hindlimbs relatively robust, tibia 0.52 of SVL and foot 0.76 of SVL; heels overlapping when hindlimbs flexed at right angles to axis of body; tibio-tarsal articulation reaching the anterior corner of eye when hindlimb is stretched along the side of the body; toes relatively long and thin, relative lengths I < II < V < III < IV; tip of each toe slightly dilated with remarkable elongated ventral callous pad, forming long and pointed disk; well-developed lateroventral grooves on toes , not meeting at the tip of disks; webbing moderate, webbing formula: I 1½ - 2⅓ II 1⅓ - 2⅓ III 1½ - 3 IV 3⅓ - 1⅔ V; presence of lateral fringes on inner and outer sides of each toes, forming distinct dermal flap on the lateral edges of toes I and V; subarticular tubercles rounded, prominent; inner metatarsal tubercle elliptic, length triple the width; outer metatarsal tubercle indistinct, small and rounded; tarsal folds and tarsal tubercle absent.

Dorsal skin of head and anterior body smooth, posterior dorsum of body rough with dense tubercles with horny spinules; week intermittent dorsolateral fold from posterior margin of upper eyelid to above groin ; upper flank with sparse tubercles; a large and smooth suprabrachial gland behind base of forelimb, not prominent; dorsal surface of upper arm smooth with sparse tubercles without spinules; the dorsal surfaces of thigh and tibia relatively rough with several weak longitudinal ridges and tubercles bearing spinules. Ventral surface of throat, body, and limbs smooth; large flattened tubercles densely arranged on the rear of thigh and around vent.

##### Coloration of holotype.

In life (Fig. 8), dorsal surface brown; pineal ocellus yellowish; mid-dorsal stripe unclear; dorsolateral fold dark brown; upper flank olive brown; lower flank creamy white; suprabrachial gland white. Dorsal limbs brown; a longitudinal black stripe on the anterior surface of the forelimb; three dark crossbars on the thigh, three on the tibia and three on the tarsus. Loreal and temporal regions dark, tympanum light brown; upper ⅓ iris brownish white and lower ⅔ iris reddish brown; maxillary gland and shoulder gland white. Throat white tinged with pink, but two subgular vocal sacs flesh colored; ventral surface of body and limbs creamy white; rear thigh tinged with pink; ventral hand flesh colored; ventral foot brown.

In preservative (Fig. 9), dorsal surface became darker; mid-dorsal stripe unclear; white spinules more distinct; pineal ocellus more distinct; crossbars on limbs became clearer; flanks and ventral surface faded.

##### Variations.

**Figure 10.**
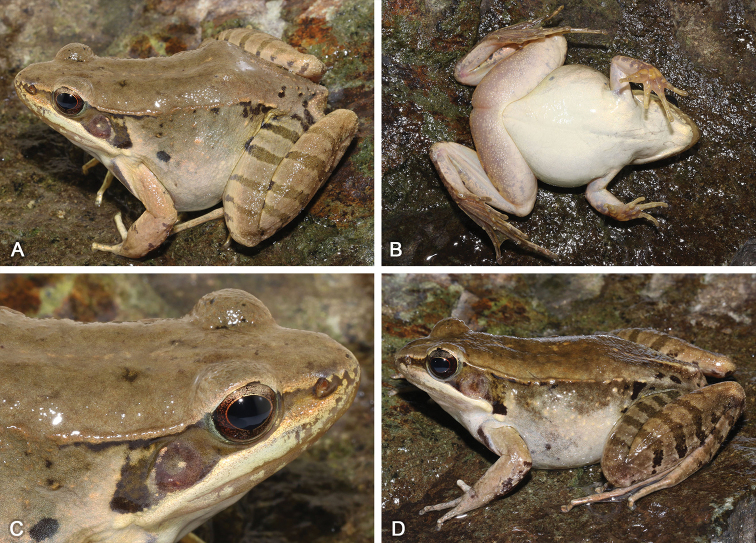
(**A**), (**B**), and (**C**) dorsolateral view, ventral view, close-up of head showing the week supratympanic fold of adult female paratype SYS a006310 of *Nidirana mangveni* sp. nov.; (**D**) adult female paratype SYS a006416.

Measurements of type series are given in Table 7. All specimens were similar in morphology. Females (62.4 ± 3.8 mm, *N* = 2) (Fig. 10) are relatively larger than males (56.2 ± 2.5 mm, *N* = 7), and more smooth than males. Pineal ocellus invisible in SYS a006310, 6311; dorsal surface light brown in SYS a006310, 6311; a short mid-dorsal stripe on the posterior dorsum in SYS a006311, 6416; spinules on the entire dorsum in SYS a006413.

**Table 7.** Measurements (in mm) of the type series of *Nidirana mangveni* sp. nov. An asterisk denotes the holotype.

	**SYS a006313** *	**SYS a006311**	**SYS a006312**	**SYS a006314 /CIB 107275**	**SYS a006413**	**SYS a006414**	**SYNU 12050569**	**SYS a006310**	**SYS a006416**
**Sex**	**Male**	**Male**	**Male**	**Male**	**Male**	**Male**	**Male**	**Female**	**Female**
SVL	54.0	58.2	56.2	53.7	59.7	57.9	53.6	59.7	65.1
HDL	20.8	21.2	20.2	20.0	22.3	21.6	21.9	21.9	24.6
HDW	18.0	19.4	18.1	18.1	20.2	20.0	18.4	19.4	19.1
SNT	7.7	7.8	7.5	7.7	7.9	8.4	8.0	8.7	8.6
IND	6.0	6.6	6.0	6.1	6.6	6.0	5.1	6.6	6.7
IOD	4.8	5.2	5.1	4.4	5.4	4.5	4.4	5.2	5.2
ED	6.3	6.5	6.4	6.4	6.1	6.3	5.6	6.4	7.0
TD	4.6	5.5	4.6	4.8	5.6	5.5	4.7	5.3	5.7
TED	1.4	1.2	1.4	1.3	1.3	1.2	1.5	1.4	1.6
HND	14.3	15.0	14.5	13.8	14.7	14.4	12.8	15.1	14.7
RAD	9.5	10.0	9.8	9.6	10.1	10.0	10.0	10.8	11.0
FTL	41.0	43.8	41.7	40.0	45.4	40.9	39.2	44.2	47.7
TIB	27.8	30.0	28.5	27.2	30.0	27.8	28.2	29.4	33.7

##### Distribution and ecology.

Currently, *Nidirana mangveni* sp. nov. is known from Mt Dapan, Mt Longmen, and Hangzhou Botanical Garden, all situated in northern Zhejiang, suggesting the *Nidirana* populations in northern Zhejiang might belong to this species. This frog inhabits natural or artificial swamps, ponds, and paddy fields. The adult males do not construct nests and calls at the water surface or the bank from May to August. The male individual SYNU12050569 which was found in early May bears indistinct nuptial pads but processes the suprabrachial gland, indicating the breeding season of this species begins from early May. The tadpoles of this species remain unknown.

##### Vocalization.

The advertisement call (*N* = 108) of *Nidirana mangveni* sp. nov. contains 2–7 repeated, identical, regular notes. The three-note call has a duration of 515.0–741.0 (684.0 ± 50.9, *N* = 26) ms; the four-note call has a duration of 722.5–1044.6 (907.0 ± 82.9, *N* = 40) ms; the five-note call has a duration of 898.1–1341.7 (1087.1 ± 108.5, *N* = 20) ms; the six-note call has a duration of 1332.0–1427.0 (1377.9 ± 26.4, *N* = 15) ms. The notes last 89.0–203.0 (136.9 ± 23.2, *N* = 462) ms with the rise time 4.1–148.6 (79.5 ± 26.9, *N* = 462) ms, and the intervals last 59.3–192.7 (116.4 ± 20.8, *N* = 354) ms.

#### Nidirana xiangica

Taxon classificationAnimaliaAnuraRanidae

Lyu & YY Wang sp. nov.

ACD64286-120B-5A6D-B9A5-E93BF5C76028

http://zoobank.org/855B3537-8FFE-408D-8062-B0D9EF3C680A

**Figure 11.**
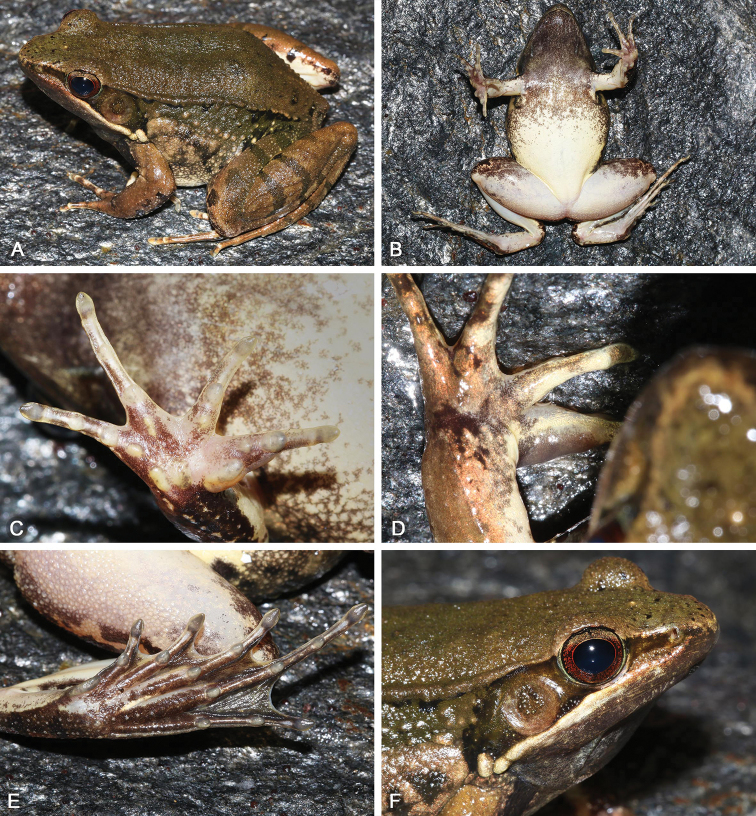
Morphological features of the adult male holotype SYS a006492 of *Nidirana xiangica* sp. nov. in life. (**A**) dorsolateral view; (**B**) ventral view; (**C**) right hand; (**D**) nuptial pad; (**E**) left foot; (**F**) close-up of head showing the dense white horny spinules on dorsum, upper eyelid, loreal region, and temporal region including tympanum.

**Figure 12.**
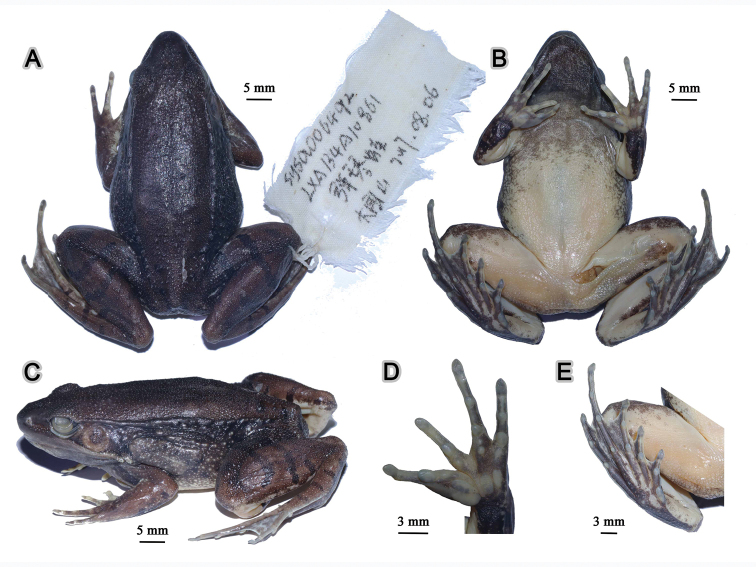
Morphological features of the adult male holotype SYS a006492 of *Nidirana xiangica* sp. nov. in preservative. (**A**) dorsal view; (**B**) ventral view; (**C**) lateral view; (**D**) left hand; (**E**) right foot.

**Figure 13.**
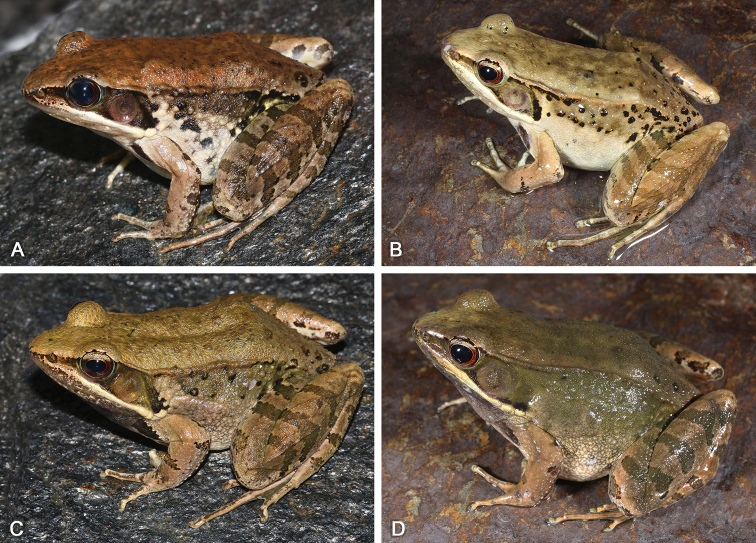
(**A**) adult female paratype SYS a006491 of *Nidirana xiangica* sp. nov.; (**B**) adult female paratype SYS a007273; (**C**) adult male paratype SYS a006493; (**D**) adult male paratype SYS a007269.

Figures 11 , 12 , 13

##### Chresonymy.

*Nidirana adenopleura*: Fei et al. 2009, 2012; Mo et al. 2014.

##### Holotype.

SYS a006492 (Figs 11, 12), adult male, collected by Zhi-Tong Lyu on 6 August 2018 from Mt Dawei (28.4237°N, 114.0793°E; ca 820 m a.s.l.), Liuyang City, Hunan Province, China.

##### Paratypes.

Nine adult specimens. Male SYS a006493/CIB 107276 and female SYS a006491, collected by Zhi-Tong Lyu and Zheng-Jiao Liu at the same time from the same locality as the holotype; male SYS a002591 and female SYS a002590, collected by Jian Zhao on 8 May 2014 from Mt Wugong (27.4079°N, 114.1671°E; ca 800 m a.s.l.), Anfu County, Jiangxi Province, China; Males SYS a 007269–7271, and females SYS a007272–7273 , collected by Zhi-Tong Lyu, Yu-Long Li, and Yao Li on 21 June 2018 from Mt Yangming (26.1155°N, 111.9591°E; ca 1150 m a.s.l.), Shuangpai County, Hunan Province, China.

##### Etymology.

The specific name *xiangica* is an adjective derived from Xiang (湘), referring to Xiangjiang River (湘江), the major drainage basin within the distribution of the new species.

##### Differential diagnosis.

*Nidirana xiangica* sp. nov. is distinguished from its congeners by the following combination of the morphological characteristics: (1) body large and elongated, with SVL 56.3–62.3 (58.0 ± 2.2, *N* = 6) mm in adult males, and SVL 53.5–62.6 (58.3 ± 4.0, *N* = 4) mm in adult females; (2) disks of digits dilated, rounded; (3) lateroventral grooves present on all digits; (4) heels just meeting; (5) tibio-tarsal articulation reaching between eye to snout; (6) mid-dorsal stripe absent; (7) dorsal surface and flanks extremely rough with dense tubercles; (8) developed supernumerary tubercles below the base of each finger, palmar tubercles prominent and distinct; (9) supratympanic fold absent; (10) white horny spinules on the entirely dorsum, dorsolateral folds, flanks, dorsal limbs, loreal region, and temporal region including tympanum in males; (11) a pair of subgular vocal sacs present; (12) one single nuptial pad on the finger I, nuptial spinules invisible; (13) suprabrachial gland large, rough and well developed, distinctly prominent; (14) calling: 2–3 notes containing a specific first note.

##### Comparison.

Morphologically, *Nidirana xiangica* sp. nov. is unique when compared with all known congeners by the combination of the following characteristics: (1) large body size, SVL 56.3–62.3 mm in males and 53.5–62.6 mm in females vs. < 53.0 mm in males or females in *N. nankunensis*, *N. okinavana*, *N. daunchina*, *N. yaoica*, *N. chapaensis*, and *N. hainanensis*; (2) relative finger lengths II < I < IV < III vs. II < I = IV < III in *N. chapaensis*; vs. II < IV < I < III in *N. leishanensis*; (3) presence of lateroventral groove on every digit vs. absent on fingers and toes in *N. pleuraden*; vs. absent or barely visible on fingers in *N. daunchina*; vs. absent on finger I in *N. guangdongensis*, *N. mangveni*, *N. adenopleura*, *N. nankunensis*, *N. okinavana*, *N. chapaensis*, and *N. lini*; (4) tibio-tarsal articulation reaches between eye to snout vs. beyond the snout tip in *N. lini*; (5) heels just meeting vs. overlapping in *N. guangdongensis*, *N. mangveni*, *N. adenopleura*, *N. nankunensis*, *N. yaoica*, *N. leishanensis*, *N. okinavana* and *N. lini*; (6) white horny spinules on the entirely dorsum, flanks, loreal region, and temporal region including tympanum in males vs. absent on dorsum and flanks or few above vent in *N. nankunensis*, *N. okinavana*, *N. daunchina*, *N. yaoica*, *N. chapaensis*, *N. leishanensis* and *N. hainanensis*; vs. present on dorsum while absent on flanks in *N. mangveni*, *N. adenopleura*, *N. lini* and *N. pleuraden*; vs. present on dorsum and flanks while absent on temporal regions in *N. guangdongensis*; (7) the presence of a single nuptial pad on finger I vs. absent in *N. hainanensis*; vs. divided into two parts in *N. chapaensis*; vs. two nuptial pads on fingers I and II respectively; (8) the presence of a pair of subgular vocal sacs vs. absent in *N. okinavana*.

##### Description of holotype.

SYS a006492 (Figs 11, 12), adult male. Body large and elongated, SVL 56.3 mm; head slightly longer than wide (HDW/HDL 0.99), flat above; snout rounded in dorsal and lateral views, slightly protruding beyond lower jaw, longer than horizontal diameter of eye (SNT/ED 1.27); canthus rostralis distinct; loreal region concave, bearing horny spinules; nostril round, directed laterally, closer to the snout than to the eye; a longitudinal swollen mandibular ridge extending from below nostril through lower edges of eye and tympanum to above insertion of arm, forming a maxillary gland and shoulder gland; supratympanic fold absent; interorbital space flat, narrower than internasal distance (IND/IOD 1.27); pupil elliptical, horizontal; temporal region including tympanum with horny spinules, tympanum distinct, round, TD/ED 0.81, and close to eye, TED/TD 0.29; pineal ocellus distinct; vomerine ridge present, bearing small teeth; tongue large, cordiform, notched behind; a pair of subgular vocal sacs present.

Forelimbs moderately robust, lower arm 0.20 of SVL and hand 0.26 of SVL; fingers thin, relative finger lengths II < I < IV < III; tip of each finger slightly dilated, forming rounded disks; lateroventral grooves on all fingers, not meeting at the tip of disks; fingers free of webbing; presence of distinct lateral fringes on inner and outer sides of fingers II, III, and IV, and on outer side of finger I; subarticular tubercles prominent and rounded; developed supernumerary tubercles below the base of each finger; three elliptic, large, prominent and very distinct palmar tubercles; a single nuptial pad on the dorsal surface of first finger, nuptial spinules invisible.

Hindlimbs relatively robust, tibia 0.50 of SVL and foot 0.74 of SVL; heels just meeting when hindlimbs flexed at right angles to axis of body; tibio-tarsal articulation reaching the loreal region when hindlimb is stretched along the side of the body; toes relatively long and thin, relative lengths I < II < V < III < IV; tip of each toe slightly dilated with remarkable elongated ventral callous pad, forming long and pointed disk; well-developed lateroventral grooves on toes , not meeting at the tip of disks; webbing moderate, webbing formula: I 1½ - 2 II 1⅓ - 2⅓ III 1⅔ - 3 IV 3⅓ - 1⅔ V; presence of lateral fringes on inner and outer sides of each toes, forming distinct dermal flap on the lateral edges of toes I and V; subarticular tubercles rounded, prominent; inner metatarsal tubercle elliptic, length triple the width; outer metatarsal tubercle indistinct, small and rounded; tarsal folds and tarsal tubercle absent.

Dorsal surface very rough with dese tubercles and dense horny spinules; developed dorsolateral fold with sparse horny spinules from posterior margin of upper eyelid to above groin but intermittent posteriorly ; flank very rough with sparse warts, dense tubercles and dense horny spinules; a large and rough suprabrachial gland behind base of forelimb, distinctly prominent; dorsal surface of forelimb rough with dense horny spinules, two weak longitudinal ridges on upper arms and slightly extending to lower arm; the dorsal surfaces of thigh and tibia rough with dese tubercles and dense horny spinules, forming several longitudinal ridges. Ventral surface of throat, body, and limbs smooth; large flattened tubercles densely arranged on the rear of thigh and around vent.

##### Coloration of holotype.

In life (Fig. 11), dorsal surface greenish brown; horny spinules on the skin white; pineal ocellus yellowish; absence of mid-dorsal stripe; dorsolateral fold greenish brown; upper flank greenish brown, warts on flank yellowish; lower flank yellowish white with black stripe; suprabrachial gland yellowish white with black stripe. Dorsal limbs brown; two greenish crossbars on the thigh, two on the tibia and three on the tarsus. Loreal and temporal regions greenish brown, tympanum light brown; upper ⅓ iris brownish white and lower ⅔ iris reddish brown; maxillary gland and shoulder gland white. Throat and anterior chest dark purplish brown; ventral surface of body and limbs creamy white; rear thigh tinged with pink; ventral hand white with large brown patches; ventral foot purplish brown.

In preservative (Fig. 12), surface of dorsum and dorsal limbs changed as dark brown; white spinules significantly distinct; crossbars on limbs clearer; ventral surface faded, throat and anterior chest dark grey.

##### Variations.

Measurements of type series are given in Table 8. All specimens were similar in morphology. Females (58.3 ± 4.0 mm, *N* = 4) (Fig. 13A, B) are not significantly larger than males (58.0 ± 2.2 mm, *N* = 6), but relatively smooth than males, not bearing white horny spinules on the dorsum, dorsolateral folds, flanks, and temporal region. Pineal ocellus invisible in SYS a006493 (Fig. 13C); dorsal surface reddish brown in SYS a006491 and greenish in SYS a007269 (Fig. 13D); numerous black spots on dorsum and flanks in SYS a007273; lateroventral grooves poorly developed on fingers I and II in SYS a002591.

**Table 8.** Measurements (in mm) of the type series of *Nidirana xiangica* sp. nov. An asterisk denotes the holotype.

	**SYS a006492** *	**SYS a006493 /CIB 107276**	**SYS a002591**	**SYS a007269**	**SYS a007270**	**SYS a007271**	**SYS a006491**	**SYS a002590**	**SYS a007272**	**SYS a007273**
**Sex**	**Male**	**Male**	**Male**	**Male**	**Male**	**Male**	**Female**	**Female**	**Female**	**Female**
SVL	56.3	62.3	57.1	57.7	56.5	57.9	53.5	56.8	62.6	60.2
HDL	20.0	23.1	19.7	21.2	22.0	22.4	19.6	21.2	24.1	22.5
HDW	19.8	22.0	19.4	20.0	19.0	20.7	18.7	20.7	22.2	18.9
SNT	8.0	9.5	8.6	8.7	8.7	8.1	7.8	8.4	9.0	8.4
IND	6.6	7.1	6.5	6.5	6.4	6.7	6.3	6.4	7.0	6.8
IOD	5.2	5.5	5.3	5.1	5.0	5.0	5.0	5.0	5.8	4.9
ED	6.3	6.8	5.9	6.3	6.0	6.6	5.9	6.4	6.2	6.0
TD	5.1	5.6	5.2	5.2	5.7	5.7	4.9	5.0	5.3	4.9
TED	1.5	1.7	1.6	1.5	1.5	1.5	1.5	1.7	1.7	1.7
HND	14.6	15.2	15.2	14.8	14.2	15.0	14.5	15.4	15.8	15.3
RAD	11.3	12.0	11.3	11.1	11.3	11.1	11.1	12.0	12.0	11.1
FTL	41.4	45.2	47.0	43.5	43.8	45.6	42.0	46.6	48.5	45.5
TIB	28.3	31.5	32.0	29.7	30.3	30.2	30.0	32.1	32.0	30.8

##### Distribution and ecology.

Currently, *Nidirana xiangica* sp. nov. is known from Mt Dawei and Mt Yangming of Hunan, Mt Wugong of western Jiangxi, and Mt Dupangling of northeastern Guangxi, indicating its potential distribution area is in the Xiangjiang River Basin. The frog inhabits natural or artificial ponds and paddy fields. This species has no behavior of nest construction, and the adult males call at the water surface from May to August. The tadpoles of this species remain unknown.

##### Vocalization.

The advertisement call (*N* = 57) of *Nidirana xiangica* sp. nov. contains 2–3 notes containing a specific first note. The two-note call has a duration of 331.9–427.0 (374.6 ± 23.5, *N* = 19) ms; the three-note call has a duration of 542.7–624.8 (569.2 ± 20.6, *N* = 38) ms. The first notes last 148.0–233.0 (170.4 ± 14.5, *N* = 57) ms with the rise time 89.8–149.1 (126.2 ± 17.5, *N* = 57) ms; the non-first notes last 60.1–128.0 (74.6 ± 11.8, *N* = 95) ms with the rise time 2.2–43.0 (27.8 ± 10.2, *N* = 95), and the intervals last 85.0–195.6 (125.8 ± 17.8, *N* = 95) ms.

## Discussion

In morphology, most anuran species seem slightly similar to each other, and within several particular species, the coloration patterns are variable among individuals. These interspecific similarities and intraspecific variabilities have caused numerous misidentifications and synonymies, and calls for comprehensive approaches in the taxonomic research on anuran frogs. For instance, the species *Nidirana guangdongensis* sp. nov. and *N. mangveni* sp. nov. overlap with each other in the morphometric comparisons, while detailed morphological comparison, phylogenetic relationships, and bioacoustics analysis reveal their differences. The species *N. xiangica* sp. nov. is significantly different from *N. adenopleura* s. s. in morphology, phylogeny, and bioacoustics, but it was previously misidentified as *N. adenopleura* possibly to deficiencies in earlier research.

The species *Nidirana adenopleura* was originally described based on several specimens from Fuhacho Village (= Maobu or Wucheng, Nantou County), central Taiwan (Boulenger 1909; Jang-Liaw and Chou 2015), and was subsequently recorded over a wide area from southern China to northern Indochina (Chan-ard et al. 1999; Fei et al. 2009, 2012). Fei et al. (2007) described the reported population of *N. adenopleura* from Mt Diaoluo, Hainan as the new species *N. hainanensis*. Chuaynkern et al. (2010) re-allocated the specimens previously identified as *N. adenopleura* from Thailand to *N. lini*. Lyu et al. (2019) recognized the population from Mt Dayao, Guangxi as the new species *N. yaoica*. These taxonomic works have indicated that the current records of *N. adenopleura* represent a species complex. In the present study, based on a comprehensive molecular, morphological, and bioacoustics analysis, the recorded populations of *N. adenopleura* from Nanling Mountains and southern Luoxiao Mountains (southern lineage), northern Zhejiang (northern lineage), and Xiangjiang River Basin (western lineage), are revealed as the new species *N. guangdongensis* sp. nov., *N. mangveni* sp. nov., and *N. xiangica* sp. nov. Currently, the recognized distribution area of the true *N. adenopleura* covers the Taiwan Island, northern Fujian, southern Zhejiang and central Jiangxi, and other reported populations beyond these areas need further study.

## Supplementary Material

XML Treatment for Nidirana guangdongensis

XML Treatment for Nidirana mangveni

XML Treatment for Nidirana xiangica

## Appendix

### Specimens examined

*Nidirana adenopleura* (29): **China: Fujian**: Yanping District: SYS a005911–5916 (topotypes of the junior synonym *N. caldwelli* (Schmidt, 1925)); Mt Wuyi: SYS a005939–5943; Jiangshi Nature Reserve: SYS a004112, 4132; Mt Yashu: SYS a005890–5891, 5901–5902; **Jiangxi**: Tongboshan Nature Reserve: SYS a001663–1665, 1667, 1698; Yangjifeng Nature Reserve: SYS a0000317, 0334; Jinggangshan Nature Reserve: SYS a004025–4027; **Zhejiang**: Jingning County: Dongkeng Town: SYS a002725–2726.

*Nidirana daunchina* (5): **China: Sichuan**: Mt Emei: SYS a004594–4595 (topotypes); Hejiang County: Zihuai Town: SYS a004930–4932.

*Nidirana hainanensis* (4): **China: Hainan**: Mt Diaoluo: SYS a003741, 7669–7671 (topotypes).

*Nidirana leishanensis* (3): **China: Guizhou**: Mt Leigong: SYS a007908 (topotypes); Mt Fanjing: SYS a007195–7196.

*Nidirana lini* (4): **China: Yunnan**: Jiangcheng County: Hongjiang Town: SYS a003967–3970 (topotypes).

*Nidirana nankunensis* (12): **China: Guangdong**: Mt Nankun: SYS a003615, 3617–3620, 4019, 4905–4907, 5717–5719 (holotype and paratypes series).

*Nidirana pleuraden* (4): **China: Yunnan**: Mt Gaoligong: SYS a003775–3778.

*Nidirana yaoica* (13): **China: Guangxi**: Mt Dayao: SYS a007009, 7011–7014, 7020–7022, NHMG 1503043–47 (holotype and paratypes series).
